# New Members of the Centrapalus Coumarin and Pauciflorin Series from *Centrapalus pauciflorus*

**DOI:** 10.3390/pharmaceutics16070907

**Published:** 2024-07-06

**Authors:** Muhammad Bello Saidu, Gordana Krstić, Petra Bombicz, Sourav De, Anita Barta, Hazhmat Ali, István Zupkó, Róbert Berkecz, Umar Shehu Gallah, Dóra Rédei, Judit Hohmann

**Affiliations:** 1 Department of Pharmacognosy, University of Szeged, Eötvös u. 6, 6720 Szeged, Hungary; mbellosaidu11@gmail.com (M.B.S.); bartaanita96@gmail.com (A.B.); redei.dora.judit@szte.hu (D.R.); 2 Faculty of Chemistry, University of Belgrade, Studentski trg 12-16, 11158 Belgrade, Serbia; gkrstic@chem.bg.ac.rs; 3 Centre for Structural Science, HUN-REN Research Centre for Natural Sciences, Magyar Tudósok körútja 2, 1117 Budapest, Hungary; bombicz.petra@ttk.hu (P.B.); de.sourav@ttk.hu (S.D.); 4 Institute of Pharmacodynamics and Biopharmacy, University of Szeged, Eötvös u. 6, 6720 Szeged, Hungary; ali.hazhmat@gmail.com (H.A.); zupko.istvan@szte.hu (I.Z.); 5 Institute of Pharmaceutical Analysis, University of Szeged, Somogyi u. 4, 6720 Szeged, Hungary; berkecz.robert@szte.hu; 6 Bioresource Department, National Research Institute for Chemical Technology (NARICT), Zaria PMB 1052, Nigeria; umarshehugallah@gmail.com; 7 HUN-REN–USZ Biologically Active Natural Products Research Group, University of Szeged, Eötvös u. 6, 6720 Szeged, Hungary

**Keywords:** *Centrapalus pauciflorus*, meroterpenoids, 5-methylcoumarins, 5-methylchromones, X-ray, antitumor effects

## Abstract

Monoterpene and 5-methylcoumarin- or 5-methylchromone-coupled meroterpenoids occurring mainly in the Asteraceae species proved to have high potency against protozoans, worms, and various tumor cells, which make them interesting targets for searching for new bioactive compounds. The African plant *Centrapalus pauciflorus* was applied in traditional medicine for healing chest pain and stomach aches. Three new meroterpenoids named centrapalus coumarin N (**2**), pauciflorins P (**3**), and Q (**4**), and the already known cyclohoehnelia coumarin (**1**), were isolated from the chloroform extract of *C. pauciflorus*, together with centrapalus coumarin O (**5**), which was obtained for the first time from a natural source. The structures were established from HRESIMS, 1D (^1^H NMR, ^13^C NMR JMOD) and 2D NMR (HSQC, HMBC, ^1^H-^1^H COSY, NOESY) spectroscopies, and the absolute stereochemistry of **5** was determined by single-crystal X-ray diffraction. Compounds **1**, **2**, and **5** are hybrid molecules of 5-methylcoumarin–monoterpene origin. Centrapalus coumarin N is the first example of meroterpenoids, where a monoterpene is fused with a coumarin and an acetophenone unit. Pauciflorins P and Q are dimeric meroterpenoid isomers. Centrapalus coumarins N and O were tested for antiproliferative activity against human adherent breast (MCF-7, MDA-MB-231), cervical (HeLa, SiHa), and ovarian (A2780) cancer cell lines, and were additionally included to obtain data concerning cancer selectivity. Both compounds exhibited moderate (IC_50_ > 10 µM) but selective activity against A2780 cells.

## 1. Introduction

The incidence of cancer is rising worldwide, making the development of more effective cancer treatments imperative from a medical standpoint. The lack of selectivity in currently available chemotherapeutic drugs is their biggest drawback, since it leads to severe side effects that frequently restrict their range of use. Therefore, the main focus of cancer research for many years has been the development of chemotherapeutical drugs with distinct modes of action that guarantee great selectivity [1].

The word meroterpenoid was introduced by Sir John W. Cornforth in an article on the biosynthesis of terpenoids [2]. Meroterpenoids can be defined as hybrid natural products derived from mixed terpenoid and polyketide or non-polyketide biosynthesis. Their carbon skeletons are formed by intramolecular cyclizations and rearrangements, resulting in different macrocyclic or polycyclic structures with a variety of functional groups. Since the introduction of the term in 1968, there has been an explosion in the number of identified compounds [3]. Meroterpenoids are widely produced by a diverse array of microorganisms, fungi, plants, and animals [4,5,6,7,8,9]. Adducts of 5-methylcoumarins or 5-methylchromones with monoterpenes have a restricted occurrence. They are mainly found in members of the tribes Vernonieae, Nassauvieae, Onoserideae, and Mutisieae in the Asteraceae family, and occasionally from a few other taxa [10,11]. Due to their restricted occurrence, only some information was published about their pharmacological activities. Among the coumarin-based meroterpenoids, 2′-epicycloisobrachycoumarinone epoxide and cycloisobrachycoumarinone epoxide, isolated from *Vernonia brachycalyx*, exhibited activity against *Leishmania major* promastigotes and *Plasmodium falciparum* schizonts in vitro and demonstrated weak inhibition on the proliferation of human lymphocytes [12]. Ethuliacoumarin A and isoethuliacoumarin A obtained from *Ethulia conyzoides* showed molluscicidal activity against *Biomphalaria glabrata* and *Bulinus truncatus*. In addition, ethuliacoumarin A was found to have cercaricidal and ovicidal potencies [13]. Among the chromane/chromene meroterpenoids, rubiginosins A–G, reported from *Rhododendron rubiginosum*, have been documented to have cytotoxic effects on various tumor cell lines (A549, HCT116, SK-HEP-1, and HL-60) [3]. Enantiomeric pairs of meroterpenoids with protein tyrosine phosphatase 1B inhibitory activity were reported in *Rhododendron fastigiatum*, with IC_50_ values ranging from 40.9 to 47.0 µM [4].

The genus *Centrapalus*, belonging to the Asteraceae family, consists of nine species, which are mainly annual or perennial herbs. Synonym names of *Centrapalus pauciflorus* (Willd.) H.Rob. include *C. galamensis* Cass., *Conyza pauciflora* Willd., *Vernonia afromontana* R.E.Fr., and *Vernonia pauciflora* (Willd.) Less., etc. *C. pauciflorus* can be found predominantly in tropical African countries [14,15]. It is a mainly unbranched, annual plant that grows 3–5 m height. In traditional African medicine, the leaves of *C. pauciflorus* are used as tea or cooked in porridge to ease chest pain and stomach aches [16]. *C. pauciflorus* was noted as a potential source of vernolic acid, a potentially useful biofeed stock. Aliphatic and phenolic acids, flavonoids, coumarins, and sesqui- and triterpenoids were previously described to be present in *C. pauciflorus* [17]. In our previous studies, 36 monoterpene-coupled meroterpenoids were isolated from the methanol extract of the leaves of *C. pauciflorus*, 28 of them were new natural compounds. The compounds belong partly to the centrapalus coumarin series that includes 5-metlylcoumarin adducts, and partly to the pauciflorin series that includes 5-methylchromone or 2,4-chromadione adducts. Among the compounds, centrapalus coumarins A, B, and F, and pauciflorin F showed remarkable antiproliferative activity (IC_50_ < 10 µM) against human adherent breast (MCF-7) and cervical (HeLa) cancer cell lines.

**Figure 1:**
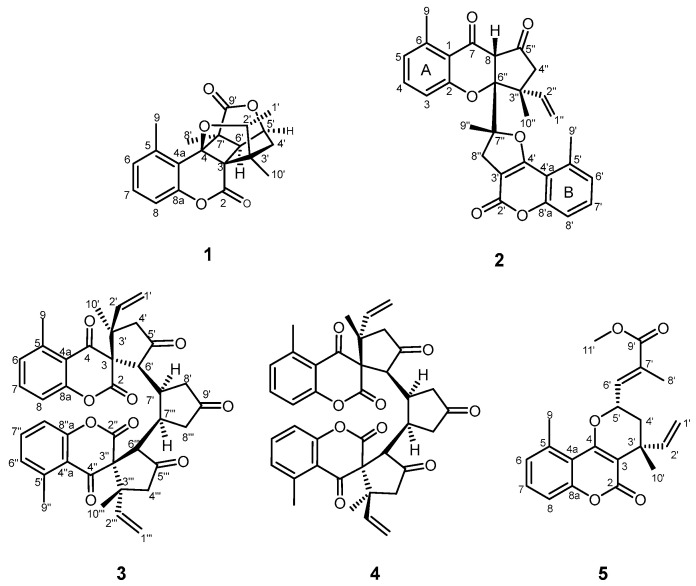
Structures of compounds **1**–**5**.

In continuation of our ongoing study on *C. pauciflorus* metabolites [16,18,19], the present paper reports the isolation and structure determination of five meroterpenoids (**1**–**5**) from the chloroform phase of the MeOH extract of leaves (Figure 1). Compounds **1**–**4** are new natural products, while compound **5** was previously reported as a semisynthetic compound. Two compounds (**2** and **5**) were investigated for antiproliferative activity against human tumor cell lines of gynecological origin and a non-malignant cell line.

## 2. Materials and Methods

### 2.1. Plant Material

The plant collection and voucher specimen depositions are reported in ref. [17].

### 2.2. General Experimental Procedures

Optical rotations were determined with a Jasco P-2000 polarimeter (Jasco International Co., Ltd., Hachioji, Tokyo, Japan). NMR spectra were recorded in CDCl_3_ on a Bruker Avance DRX 500 spectrometer (Billerica, MA, USA) at 500 MHz (^1^H) and 125 MHz (^13^C). The signals of the deuterated solvent [7.260 ppm (^1^H) and 77.16 ppm (^13^C)] were taken as references. Two-dimensional (2D) experiments were performed with TopSpin 3.6.1 Bruker software. In the correlation spectroscopy (^1^H-^1^H COSY), nuclear Overhauser effect spectroscopy (NOESY), heteronuclear single quantum coherence spectroscopy (HSQC), and heteronuclear multiple bond correlation (HMBC) experiments, gradient-enhanced versions were applied. HRESIMS spectra were acquired on a Thermo Scientific Q-Exactive Plus Orbitrap (Waltham, MA, USA) mass spectrometer equipped with an ESI ion source in a positive ionization mode. MassLynx 4.1 (SCN805) software was used for data acquisition and processing. Chem3D 20.1.1. software was applied to calculate energy-minimized 3D structures. For the separation of the extract and fractions, vacuum liquid chromatography (VLC) was carried out on silica gel [15 μm, Merck (Budapest, Hungary)]; the LiChroprep RP-18 (40–63 μm, Merck) stationary phase was applied for reversed-phase (RP) VLC; and open column chromatography (CC) was accomplished on polyamide [MP Biomedicals (Irvine, CA, USA), 50–160 µm]. Preparative thin-layer chromatography (PTLC) was performed on silica gel 60 F_254_ plates (Merck). HPLC was carried out on WUFENG (Shanghai, China), WATERS (Milford, MA, USA), and Agilent (Santa Clara, CA, USA) HPLC instruments using normal-phase (NP) LiChrospher Si 60 (250 mm × 4 mm, 5 µm) and Luna (R) Silica (2) 100 (250 mm × 21.2 mm, 5 µm), as well as RP Kinetex C_18_ 100 A (150 mm × 4.6 mm, 5 μm) (Phenomenex, Torrance, CA, USA) and Agilent ZORBAX ODS C_18_ 100A (250 mm × 9.4 mm, 5 µm) (Santa Clara, CA, USA) columns. TLC plates were detected under a UV light at 254 nm and sprayed with concentrated sulfuric acid, followed by 5 min of heating. All solvents used for CC and TLC were of at least analytical grade (VWR Ltd., Debrecen, Hungary).

### 2.3. Extraction and Isolation

Fractions A–I were obtained from the chloroform phase of MeOH extract of *C. pauciflorus* by open column chromatography (OCC) and vacuum liquid chromatography (VLC), as described in ref. [19]. Fractions B and C eluted with cyclohexane–EtOAc–EtOH (8:2:0, 50:20:1.5, and 50:20:3) were further chromatographed on NP- and RP-VLC as follows (Figure S43). The RP-VLC of fraction B using MeOH-H_2_O mixtures (from 40:60 to 90:10) as eluents afforded three subfractions (B/I–III). Subfraction B/III underwent NP-VLC separation with a gradient system of cyclohexane–EtOAc (from 98:2 to 80:20), yielding fractions B/III/1–2. Furthermore, NP-HPLC purification of fraction B/III/2 using an isocratic system of *n*-hexane–EtOAc–MeOH (98:1:1) as the mobile phase afforded four fractions (B/III/2/a–d). Then, RP-HPLC of fraction B/III/2/d was carried out with the MeOH–H_2_O isocratic system (75:25) as eluents to gain pure compound **5** (5.0 mg; t_R_ = 18.2 min). Fraction C was subjected to reverse phase Si gel flash column chromatography (RP-FC), with MeOH-H_2_O mixtures (from 10:90 to 100:0) as eluents. Seven subfractions were obtained, namely C/I–VII. Subfraction C/III was subjected to NP-VLC with the *n*-hexane–CHCl_3_ (from 90:10 to 20:80) gradient solvent system, which afforded fractions C/III/1–5. Fraction C/III/1 was chromatographed by NP-VLC using cyclohexane–EtOAc mixtures (from 98:2 to 80:20) as mobile phase yielding subfractions (C/III/1/a–g). Fraction C/III/1/g was separated by NP-HPLC using *n*-hexane–EtOAc–MeOH (80:19:1) as the mobile phase to obtain four subfractions (C/III/1/g/1–4). The final purification of fractions C/III/1/g/2 was performed by RP-HPLC, with MeOH–H_2_O (75:25) as the eluent to create compound **1** (0.8 mg; R_f_ = 0.63) in a pure form. Fraction C/IV was subjected to NP-VLC separation using mixtures of *n*-hexane–CHCl_3_ (from 60:40 to 10:90) as a mobile phase, which afforded fractions C/IV/1–3. NP-VLC separation of fraction C/IV/1 with a mobile phase of the cyclohexane–EtOAc gradient system (from 98:2 to 80:20) yielded the fractions C/IV/1/a–c. Repeated NP-HPLC analysis of fraction C/IV/1/c with an elution of *n*-hexane–EtOAc–MeOH (80:19:1) led to the separation of fractions C/IV/1/c/1–5. The final purification of C/IV/1/c/1 was made by RP-HPLC (mobile phase MeOH–H_2_O 8:2), which resulted in the isolation of compound **2** (1.9 mg; t_R_ = 5.7 min). NP-VLC separation using the mobile phase of *n*-hexane–CHCl_3_ (from 60:40 to 10:90) was conducted on fraction C/V. This separation yielded three subfractions (C/V/1–3). Fraction C/V/1 was subjected to the next NP-VLC chromatography (mobile phase cyclohexane–EtOAc from 98:2 to 80:20) affording fractions C/V/1/a–d. In the final purification steps, fraction C/V/1/d was subjected to NP-HPLC using *n*-hexane–EtOAc–MeOH (80:19:1) and then the main fraction to RP-HPLC (mobile phase MeOH–H_2_O 67:33) to yield compounds **3** (0.9 mg; t_R_ = 26 min) and **4** (0.8 mg; t_R_ = 29 min).

### 2.4. Cyclohoehnelia Coumarin (**1**)

White amorphous powder; [α]_D_^25^ +98.6 (*c* 0.05, CHCl_3_); ^1^H and ^13^C NMR data, see Figures S3 and S4; positive-ion HRESIMS *m*/*z* 341.1385 [M + H]^+^ (calcd for C_20_H_21_O_5_^+^ 341.1384).

### 2.5. Centrapalus Coumarin N (**2**)

White amorphous powder; [α]_D_^27^ −83.7 (*c* 0.1, CHCl_3_); ^1^H and ^13^C NMR data, see Table 1; positive-ion HRESIMS *m*/*z* 471.1804 [M + H]^+^ (calcd for C_29_H_27_O_6_^+^ 471.1802).

### 2.6. Pauciflorin P (**3**)

White amorphous powder; [α]_D_^25^ −206.8 (*c* 0.05, CHCl_3_); ^1^H and ^13^C NMR data, see Table 2; positive-ion HRESIMS *m/z* 649.2441 [M + H]^+^ (calcd for C_39_H_37_O_9_^+^ 649.2432).

### 2.7. Pauciflorin Q (**4**)

White amorphous powder; [α]_D_^25^ +105.1 (*c* 0.05, CHCl_3_); ^1^H and ^13^C NMR data, see Table 2; positive-ion HRESIMS *m*/*z* 671.2244 [M + Na]^+^ (calcd for C_39_H_36_O_9_Na^+^ 671.2252).

### 2.8. Centrapalus Coumarin O (**5**)

White crystal obtained from the mixture of MeOH and EtOAc (3:2); ^1^H and ^13^C NMR data, see Table 1; positive-ion HRESIMS *m*/*z* 355.1538 [M + H]^+^ (calcd for C_21_H_23_O_5_^+^ 355.1541).

### 2.9. X-ray Crystallography of Centrapalus Coumarin O (**5**)

The absolute configuration of **5** was determined by X-ray crystallography. The single crystal was harvested from a solution prepared with the mixture of MeOH and ethyl acetate in a 3:2 ratio by evaporation. Single-crystal X-ray diffraction data collection was performed on well-developed, colorless, clear, transparent, block-type crystals of **5** using CuKα radiation. Compound **5** crystallizes in the orthorhombic crystal system, in the chiral space group *P*2_1_2_1_2_1_ (#19). The absolute configurations of the chiral atoms are C-3′ (*R*) and C-5′ (*S*). Details of molecular conformation, intra- and intermolecular interactions, and packing arrangement are published as Supporting Information.

Crystallographic data of compound **5** have been deposited in the Cambridge Crystallographic Data Centre with the deposition number 2314286. Copies of the data can be obtained free of charge via www.ccdc.cam.ac.uk/data_request/cif, by emailing data_request@ccdc.cam.ac.uk, or by contacting the Cambridge Crystallographic Data Centre, 12 Union Road, Cambridge CB2 1EZ, UK [fax: +44-(0)1223-336033].

Crystallographic data of (3′*R*),(5′*S*)-(*E*)-methyl 3-(4,10-dimethyl-5-oxo-4-vinyl-2,3,4,5-tetrahydropyrano[3,2-c]chromen-2-yl)-2-methyl acrylate (**5**): Intensity data were collected on a ‘Rigaku RAXIS-RAPID II’ diffractometer (graphite monochromator; Cu-Kα radiation, λ = 1.54187 Å) at 293(2) K in the range of 4.371 ≤ θ ≤ 68.167 [20]. A total of 24,816 reflections was collected, of which 3424 were unique [*R*(int) = 0.0374, *R*(σ) = 0.0223]; intensities of 3084 reflections were greater than 2σ(I). Completeness is θ = 0.996. A numerical absorption correction was applied to the data (the minimum and maximum transmission factors were 0.931 and 0.973, respectively). The structure was solved by direct methods [20]. Anisotropic full-matrix least-squares refinement [21] on F^2^ for all non-hydrogen atoms yielded *R*1 = 0.0437 and *wR*2 = 0.1050 for 1332 [I > 2σ(I)] and *R*1 = 0.0489 and *wR*2 = 0.1081 for all (3424) intensity data (number of parameters = 249, goodness-of-fit = 1.097, and the maximum and mean shift/esd are 0.000 and 0.000). The absolute structure parameter is 0.09(6) (Friedel coverage: 0.742, Friedel fraction max.: 0.995, and Friedel fraction full: 0.995). The maximum and minimum residual electron densities in the final difference map were 0.161 and −0.131e.Å^−3^. The weighting scheme applied was w = 1/[σ^2^(*F*_o_^2^) + (0.0589*P*)^2^ + 0.0865*P*], where *P* = (*F*_o_^2^ + 2*F*_c_^2^)/3. Hydrogen atomic positions were located in difference maps, then they were constrained. Hydrogen atoms were included in structure factor calculations, but they were not refined. The isotropic displacement parameters of the hydrogen atoms were approximated from the *U*(eq) value of the atom they were bonded to.

### 2.10. Determination of Antiproliferative Activities

The antiproliferative activities of the isolated compounds were investigated on a panel of human adherent cancer cell lines using the 3-(4,5-dimethylthiazol-2-yl)-2,5-diphenyltetrazolium bromide) (MTT) assay [22]. Cell lines isolated from cervical (HeLa and SiHa), breast (MCF-7 and MDA-MB-231), ovarian cancers (A2780), and non-malignant mouse fibroblasts (NIH/3T3) were purchased from the European Collection of Cell Cultures (Salisbury, UK). The SiHa cell line was obtained from the American Tissue Culture Collection (Manassas, VA, USA). The cell culturing and determination of the antiproliferative effects of the isolated compounds were conducted using a methodology described previously [15]. Calculations were performed using GraphPad Prism 5.01 software (GraphPad Software Inc., San Diego, CA, USA).

## 3. Results and Discussion

### 3.1. Isolation and Structure Elucidation of Compounds

The chloroform phase of the MeOH extract, prepared from *C. pauciflorus* leaves, was fractionated by open column chromatography on polyamide yielding five fractions. The chloroform extract and its fractions were tested for antiproliferative activity on human cancer cell lines of gynecological origin [MCF-7, MDA-MB-231 (breast), HeLa, SiHa (cervical), and A2780 cells (ovarian)]. The fraction eluted with 60% MeOH displayed a 63.7–85.3% inhibition at 30 µg/mL [17]. This fraction was studied in detail and subjected to multistep chromatographic purification (Figure S43). Five compounds (**1**–**5**) (Figure 1) were isolated, and their structures were elucidated using HRESIMS and 1D (^1^H and ^13^C JMOD) and 2D NMR (^1^H-^1^H COSY, HSQC, HMBC, and NOESY) experiments.

Compound (**1**) was isolated as a white amorphous powder with an optical rotation of [α]_D_^25^ +98.6 (*c* 0.05, CHCl_3_). The molecular ion at *m*/*z* 341.1385 [M + H]^+^ (calcd for C_20_H_21_O_5_^+^ 341.1384) in HRESIMS indicated a molecular composition C_20_H_20_O_5_. The ^1^H NMR, ^13^C NMR JMOD spectral data supported by HMBC, HSQC, ^1^H-^1^H COSY, and NOESY spectra were in good agreement with those of cyclohoehnelia coumarin (**1**) isolated by Schuster et al. from *Ethulia vernonioides* [23]. This compound is a coumarin monoterpene adduct, a [2+2] cycloaddition product of hoehnelia coumarin.

**Figure 2:**
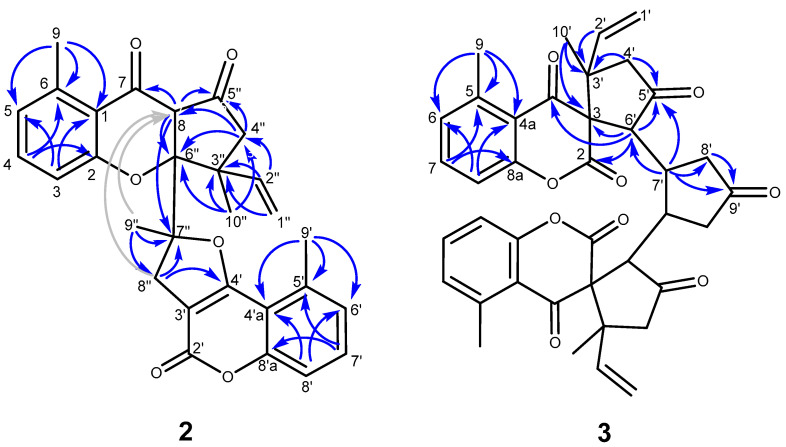
HMBC correlations of compounds **2** and **3** (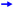 two- and three-bond correlations; 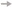 four-bond correlation).

Centrapalus coumarin N (**2**) was isolated as a white amorphous powder with an optical rotation of [α]_D_^27^ −83.7 (*c* 0.1, CHCl_3_). The molecular formula of **2** was found to be C_29_H_26_O_6_ from the peak observed at *m*/*z* 471.1804 [M + H]^+^ (calcd for C_29_H_27_O_6_^+^ 471.1802) in the HRESIMS spectrum. The ^1^H NMR and ^13^C NMR JMOD spectra of **2** indicated that this compound is composed of 5-methylcoumarin and 2-hydroxy-6-methyl-acetophenone and a monoterpene unit (Table 1). The ^1^H-^1^H COSY spectrum of **2** exhibited the presence of two 1,2,3-trisubstituted aromatic rings [A-ring: *δ*_H_ 6.14 d (*J* = 7.9 Hz), 6.86 t (*J* = 7.9 Hz), 6.48 d (*J* = 7.9 Hz); B-ring: *δ*_H_ 6.77 d (*J* = 7.9 Hz), 7.18 t (*J* = 7.9 Hz), and 6.94 d (*J* = 7.9 Hz) and a vinyl group [*δ*_H_ 5.01, d (17.3 Hz), 4.93, d (10.8 Hz), 5.60, dd (17.3, 10.8 Hz)]. The 5-methylcoumarin part coupled with the monoterpene part at C-3′ and C-4′ was calculated based on similar NMR data with those of previously published centrapalus coumarins [16] and HMBC correlations of aromatic ring B (Figure 2).

**Figure 3:**
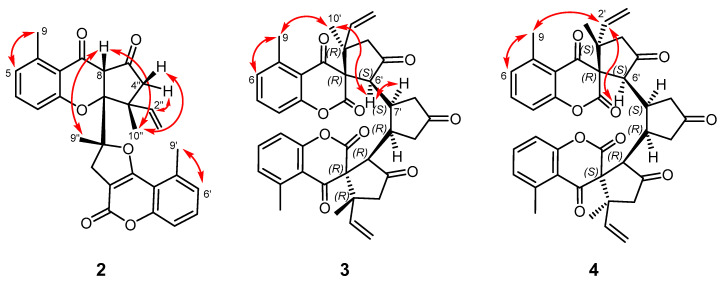
Key NOESY correlations (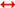) of compounds **2**, **3**, and **4**.

Further construction of the molecule was carried out by the inspection of the HMBC correlations of quaternary carbons. Long-range heteronuclear correlations of H_2_-8″ with C-3′, C-4′, and C-7″, and H_3_-9″ with C-7″ indicated a methyl-substituted dihydrofuran ring condensed with the coumarin skeleton. The structural fragment of the monoterpene unit –CO–CH_2_–C(CH_3_)CH=CH_2_ [C-5″–C-4″–C-3″(C-10″)–C-2″–C-1″] was elucidated with the aid of HMBC cross-peaks between H_2_-1″, H-2″, H_2_-4″, H_3_-10″, and C-3″, and between H_2_-4″ and C-5″. The remaining quaternary carbon (*δ*_C_ 96.0) and methine groups (*δ*_H_ 3.78 s; *δ*_C_ 64.0) were assigned to C-6″ and C-8, respectively, as indicated by the two- and three-bond correlations of H_2_-4″ and H_3_-10″ with C-6″, and those of H-8 with C-7, C-5″, C-6″, and C-7″ (Figure 2). The above data establish the constitution of this compound as depicted in the structural formula of **2**. The relative stereochemistry of chiral centers C-8, C-3″, C-6″, and C-7″ was determined based on a NOESY measurement. Starting from the *β*-position of H-8, Overhauser effects between H-8/H_3_-9″ and H-8/H_3_-10″ indicated the *β*-orientation of these methyls. Accordingly, NOESY correlations between H_3_-10″/H-4″*β* (*δ*_H_ 2.46 d) and H-2″/H-4″*α* (*δ*_H_ 2.98 d) were observed (Figure 3). This stereochemistry was consistent with the measured atom distances in the energy-minimalized 3D structure of **2** (Figure S1). NOE effects between H_3_-9/H-5 and H_3_-9′/H-6′ corroborated the positions of the methyl groups on the aromatic rings. The relative configuration of **2** was established as 8S*,3″R*,6″R*,7″R*.

Pauciflorin P (**3**) was isolated as a white amorphous powder with an optical rotation of [α]_D_^25^ −206.8 (*c* 0.05, CHCl_3_). The molecular formula of **3** was deduced from the observed peak at *m*/*z* 649.2441 [M + H]^+^ (calcd for C_39_H_37_O_9_^+^ 649.2432) in the positive-ion HRESIMS. The molecular formula suggested a dimeric compound; the molecular mass was almost double that of other members of the pauciflorin series. The ^1^H and ^13^C JMOD NMR data indicated a symmetric dimer because only half of the protons and carbons were detectable in the spectra (Table 2). A 5-methyl-2,4-chromadione part was evident based on similar ^1^H and ^13^C NMR signals with those of pauciflorin O. Furthermore, the H-1′–H-5′ structural fragment of the monoterpene part was also found to be identical to that of pauciflorin O [19]. The ^1^H-^1^H COSY spectrum confirmed the sequences of correlated protons at *δ*_H_ 3.46 d, 2.53 m, 1.67 m, and 2.89 m (–CH–CH–CH_2_–). This molecular part was presented as fragment C-6′–C-7′–C-8′, with respect to the HMBC correlations of H-6 with C-2, C-3, C-4, C-4′, and C-7′; H-7′ with C-8′ and C-9′; and H_2_-8′ with C-9′ (Figure 2). H_3_-10′ correlated with C-2′, C-3′, and C-3, referring to its position at C-3′. The 10′-methyl group was placed at C-3′ as. The structure elucidated by this method in combination with its mirror image structure afforded the entire molecule **3**. The key NOESY correlation for determining the relative stereochemistry was detected between H_3_-9 (*δ*_H_ 2.62 s) and H_3_-10′ (*δ*_H_ 0.95 s). According to 3D modeling, this correlation is possible, only if the *α*-oriented 10′-methyl group is connected to the spiro-structure, as presented in structural formula **3** [19]. Further correlations of H_3_-10′ with H-4′*α* (*δ*_H_ 2.10 d), and H-4′*α* with H-6′ (*δ*_H_ 3.46 d) determined their *α*-orientation. The Overhauser effect between H-6′ and H-7′ (*δ*_H_ 2.53 m) indicated their α orientation. The NOESY correlations of H-6′/H-8′*α*, H_3_-10′/H-4′*α*, and H-2′/H-4′*β* also afforded the stereochemical assignment of methylene protons (Figure 3 and Figure S2). According to the above conclusions, the relative configuration of **3** is 3R*,3′R*,6′S*,7′S*,3″R*,3‴R*,6‴S*,7‴R*.

Pauciflorin Q (**4**), a white amorphous powder with optical rotation of [α]_D_^25^ +105.1 (*c* 0.05, CHCl_3_), was determined to have the same molecular formula as that of pauciflorin P (**3**), based on the presence of the sodiated molecular ion peak at *m*/*z* 671.2244 [M + Na]^+^ (calcd for C_39_H_36_O_9_Na^+^ 671.2252) in the HRESIMS spectrum. ^1^H and ^13^C NMR JMOD data (Table 2) are also very similar, suggesting that this compound is also a symmetric dimeric compound. Notable differences were recorded in the chemical shifts of H_2_-1′ [**4**: *δ*_H_ 5.05 d (*J* = 17.2 Hz), 4.84 d (*J* = 10.8 Hz); **3**: *δ*_H_ 4.82 d (*J* = 17.2 Hz), 4.86 d (*J* = 10.7 Hz)], H_2_-4′ [**4**: *δ*_H_ 2.51 d, 2.43 d (each *J* = 18.6 Hz); **3**: *δ*_H_ 2.10 d, 2.80 d (each *J* = 18.6 Hz)], H_3_-10′ (**4**: *δ*_H_ 1.07 s; **3**: *δ*_H_ 0.95 s), C-1′ (**4**: *δ*_C_ 114.9; **3**: *δ*_C_ 116.8), C-2′ (**4**: *δ*_C_ 140.3; **3**: *δ*_C_ 138.8), and C-10′ (**4**: *δ*_C_ 21.6; **3**: *δ*_C_ 23.8). This indicates that compounds **3** and **4** differ in the configuration of C-3′. NOESY correlations between H-2′ and H_3_-9, and between H-2′ and H-6′ confirmed the *α*-position of the vinyl group and *β*-position of the 10′-methyl group (Figure 3), allowing the assignment of the relative configuration of **4** as 3R*,3′S*,6′S*,7′S*,3″R*,3‴S*,6‴S*,7‴R*.

Centrapalus coumarin O (**5**), a white crystal, is a new natural product. Previously, it was semisynthetized from a compound of *Ethulia conyzoides* and obtained by methylation as a mixture of 5′*α* and 5′*β* isomers [24]. ^1^H and ^13^C NMR assignments of compound **5** were determined from 2D NMR spectra. The NMR data of **5** are listed in Table 3, because in a previous publication, only ^1^H NMR chemical shifts were published for the mixture of the isomers. 

**Figure 4:**
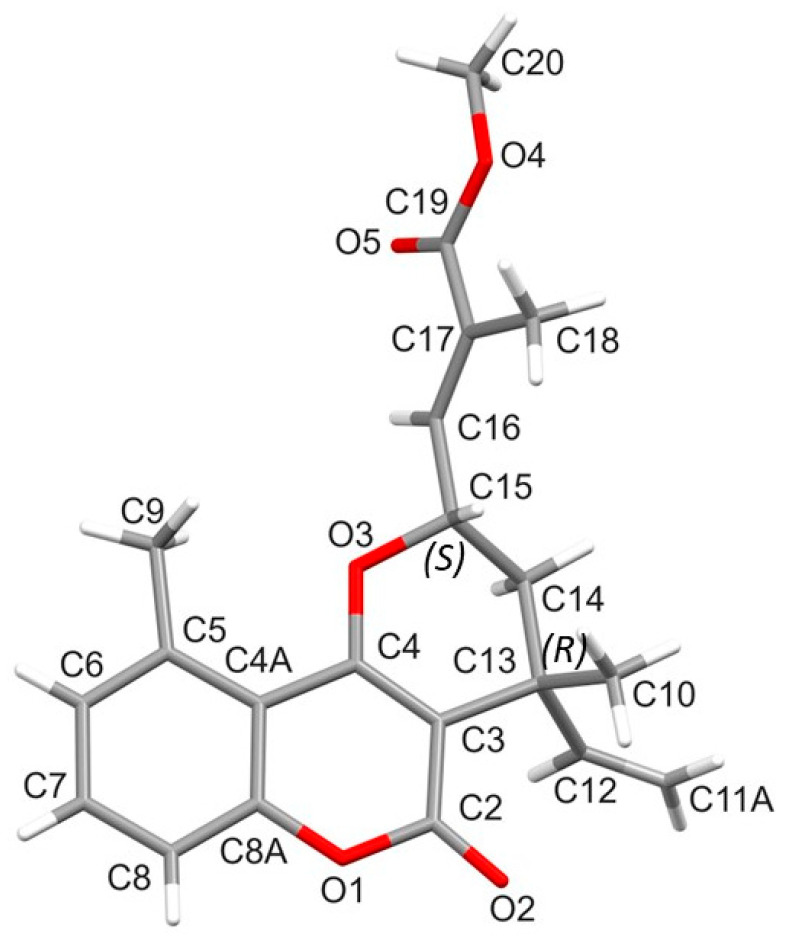
Molecular structure diagram of compound **5**.

The absolute configuration of **5** was analyzed by single-crystal X-ray diffraction, and absolute configurations of C-3′ and C-5′ were established as *R* and *S*, respectively (Figure 4). The Flack parameter was 0.09(6).

### 3.2. Antiproliferative Activity of Centrapalus Coumarins N and O

The antiproliferative properties of centrapalus coumarins N (**2**) and O (**5**) were investigated against a panel of human adherent tumor cell lines of gynecological origin by in vitro MTT assay (Table 4). The amounts of the other compounds were insufficient for the in vitro assay. Compounds **2** and **5** were tested on the breast (MCF-7, MDA-MB-231), cervical (HeLa, SiHa), and ovarian (A2780) cancer cells in two concentrations (10 and 30 µM). The anticancer agent cisplatin was used as a positive control. The A2780 ovarian cell line appeared to be the most sensitive (growth inhibition > 50%); therefore, IC_50_ values were determined for the compounds (IC_50_ **2**: 20.11 µM, **5**: 15.99 µM). Non-cancerous mouse fibroblasts (NIH/3T3) were additionally included to characterize the cancer selectivity of the tested compounds. Since compounds **2** and **5** elicited weak action against fibroblasts, lower than against the ovarian cancer cells, their effect detected on A2780 cells could be regarded as selective.

**Figure 5:**
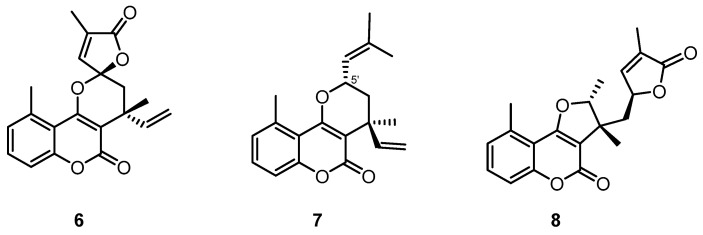
Structures of compounds **6**–**8**.

Since meroterpenoids are a structurally outstandingly diverse group of natural products, elucidating their structure–activity relationships is a great challenge. Based on our previous works, we can make a few statements regarding structure–activity relationships. Comparing the structures of 5-methylcoumarin derivative **6** (Figure 5) previously isolated from *C. pauciflorus* [16] and centrapalus coumarin O (**5**), it can be observed that, in position 5′, compound **5** contains a C_4_ unit 2-carboxymethyl-2-methyl-1-propenyl group, which forms a lactone ring in **6**. Since the antiproliferative activity of **6** is more pronounced on MCF-7 (IC_50_ 15.30 µM), HeLa (IC_50_ 14.59 µM), and SiHa (IC_50_ 18.94 µM) cells than those of **5**, it can be stated that the lactone structure at C-5′ is preferable for the effect on breast and cervical cancer cells. However, compound **5** exerted greater activity against ovarian cancer cells (A2780) (IC_50_ 15.99 µM) than **6** (IC_50_ of 29.94 µM). Centrapalus coumarin A (**7**) with the *α*-dimethylvinyl group at C-5′ showed a similar effect against the ovarian cancer cell line (IC_50_ 19.65 µM) as **5**; in addition, it was also potent against HeLa cells (IC_50_ of 9.21 µM). 

Centrapalus coumarin N (**2**) contains a 5-methylchromone and a 5-methylcoumarin part; the latter structure can also be found in the *C. pauciflorus* metabolite Hoehnelia coumarin (**8**), where it is connected to a methyl-dihydrofuranolactone ring. As the Hoehnelia coumarin (**8**) was ineffective in our previous antiproliferative assay [16], the activity of centrapalus coumarin N (**2**) against A2780 ovarian cancer cells (IC_50_ 20.11 µM) most probably can be attributed to the 5-methylchromone part of the molecule.

Furthermore, it was observed that a hydroxy function on the aromatic ring may substantially increase the antiproliferative activity. This was found when the antiproliferative activities of pauciflorin F and pauciflorin G were compared [19]. A set of rubiginosins and anthopogochromene B, all molecules containing a hydroxy group on the coumarin core, were reported to exert moderate cytotoxic activity against four cancer cell lines [25]. In addition to the above, the compounds are worth investigating further. Their other activities reported for monoterpene-based meroterpenoids include antioxidant, antimicrobial, and various enzyme inhibitory effects [4].

## 4. Conclusions

The chloroform phase of the methanol extract from *C. pauciflorus* leaves underwent a multistep isolation process with the goal of identifying new plant metabolites with potential antiproliferative activity. Five compounds (**1**–**5**) were reported in the present paper that represent structural novelties: centrapalus coumarins N (**2**) and O (**5**) isolated from *C. pauciflorus* are hybrid molecules with 5-methylcoumarin–monoterpene origin. Centrapalus coumarin N (**2**) is the first example of a meroterpenoid where the monoterpene is fused with a coumarin and an acetophenone unit. Pauciflorins P (**3**) and Q (**4**) are dimeric meroterpenoid isomers with a symmetric structure.

The identified structures allow the recognition of some biogenetic relationships. Compounds **3** and **4** can be derived by coupling two pauciflorin N molecules, after rearrangement, and the loss of a C_1_ unit. Usually, the terpenoid unit of meroterpenoids follows the isoprene rule [e.g., centrapalus coumarin O (**5**)]; however, in compounds **2**–**4**, it is not possible to sequence the isoprene subunits according to the rule because of a rearrangement. 

The tested compounds, centrapalus coumarins N (**2**) and O (**5**), proved to have considerable and selective antiproliferative activity against ovarian cancer cells. Moreover, semisynthetic modifications can result in structural analogs that can have better activities on tumor cell lines. On the other hand, meroterpenoids exhibit remarkable structural diversity, and their biological activities are not restricted to the inhibition of the proliferation of malignant cells. Compounds of this class with antimicrobial, anti-inflammatory, nephroprotective, neuroprotective, and enzyme inhibitory activities (e.g., cyclooxygenase, acetylcholinesterase, butyrylcholinesterase, protein tyrosine phosphatase 1B, and protein farnesyl transferase) have been reported [4,26]. Based on this consideration, the currently presented set of meroterpenes may exert therapeutically relevant actions on different targets, making them valuable tools for drug discovery.

## Figures and Tables

**Table 1:** ^1^H and ^13^C NMR data of compound **2** [*δ* ppm (*J* = Hz), CDCl_3_, 500 MHz (^1^H), and 125 MHz (^13^C)].

Position	^1^H	^13^C
1	-	119.1, C
2	-	170.8, C
3	6.48, d (7.9)	109.2, CH
4	6.86, t (7.9)	137.2, CH
5	6.14, d (7.9)	123.3, CH
6	-	138.9, C
7	-	198.9, C
8	3.78, s	64.0, CH
9	2.41, s	17.3, CH_3_
2′	-	160.4, C
3′	-	102.8, C
4′	-	165.6, C
4′a	-	111.0, C
5′	-	136.0, C
6′	6.77, d (7.9)	125.7, CH
7′	7.18, t (7.9)	131.2, CH
8′	6.94, d (7.9)	114.5, CH
8′a	-	155.5, C
9′	2.30, s	21.1, CH_3_
1″a	5.01, d (17.3)	116.8, CH_2_
1″b	4.94, d (10.8)	
2″	5.60, dd (17.3, 10.8)	137.5, CH
3″	-	47.5, C
4″*α*	2.98, d (18.0)	52.0, CH_2_
4″*β*	2.46, d (18.0)	
5″	-	210.7, C
6″	-	96.0, C
7″	-	92.2, C
8″*α*	3.32, d (15.3)	37.6, CH_2_
8″*β*	2.83, d (15.3)	
9″	1.83, s	30.4, CH_3_
10″	1.36, s	21.0, CH_3_

**Table 2:** ^1^H and ^13^C NMR data of compounds **3** and **4** [*δ* ppm (*J* = Hz), CDCl_3_, 500 MHz (^1^H), and 125 MHz (^13^C)].

Position	3	4	3	4
2, 2″	-	-	167.5, C	167.7, C
3, 3″	-	-	68.8, C	68.9, C
4, 4″	-	-	189.9, C	189.6, C
5, 5″	-	-	141.5, C	141.9, C
6, 6″	7.00, d (8.0)	7.05, d (7.8)	127.4, C	127.7, C
7, 7″	7.24, t (8.0)	7.29, t (7.8)	134.5, CH	134.5, CH
8, 8″	6.34, d (8.0)	6.43, d (7.8)	115.0, CH	115.0, CH
8a, 8″a	-	-	155.2, CH	155.1, CH
4a, 4″a	-	-	118.5, C	118.9, C
9	2.62, s	2.58, s	21.9, CH_3_	21.7, CH_3_
1′, 1‴	4.82, d (17.2)	5.05, d (17.2)	116.8, CH_2_	114.9, CH_2_
	4.86, d (10.7)	4.84, d (10.8)		
2′, 2‴	5.51, dd (17.2, 10.7)	5.52, dd (17.2, 10.8)	138.8, CH	140.3, CH
3′, 3‴	-	-	48.9, C	48.8, C
4′, 4‴	2.10, d (18.6) (*α*)	2.43, d (18.6) (*α*)	47.6, CH_2_	46.9, CH_2_
	2.80, d (18.6) (*β*)	2.51, d (18.6) (*β*)		
5′, 5‴	-	-	212.2, C	212.2, C
6′, 6‴	3.46, d (9.1)	3.40, d (8.9)	52.9, CH	53.7, CH
7′, 7‴	2.53, m	2.48, m	45.3, CH	45.2, CH
8′, 8‴	1.67, m (*α*)	1.68, m (*α*)	30.1, CH_2_	30.1, CH_2_
	2.89, m (*β*)	2.86, m (*β*)		
9′	-	-	221.0, C	221.3, C
10′, 10‴	0.95, s	1.07, s	23.8, CH_3_	21.6, CH_3_

**Table 3:** ^1^H and ^13^C NMR data of compound **5** [*δ* ppm (*J* = Hz), CDCl_3_, 500 MHz (^1^H), and 125 MHz (^13^C)].

Position	^1^H	
2	-	162.7, C
3	-	108.0, C
4	-	160.6, C
4a	-	114.5, C
5	-	137.0, C
6	7.01, d (7.8)	127.6, CH
7	7.33, t (7.8)	131.0, CH
8	7.14, d (7.8)	115.0, CH
8a	-	153.9, C
9	2.65, s	23.8, CH_3_
1′	5.13, d (10.6) 5.15, d (17.5)	112.8, CH_2_
2′	6.18, dd (17.5, 10.6)	144.4, CH
3′	-	36.5, C
4′*α*	2.04, dd (14.1, 11.9)	41.4, CH_2_
4′*β*	1.80, dd (14.1, 1.9)	
5′	5.04, ddd (11.9, 7.8, 1.3)	72.1, CH
6′	6.89, dd (7.8, 1.3)	137.7, CH
7′	-	131.1, C
8′	1.97, d (1.3)	13.3, CH_3_
9′	-	167.7, C
10′	1.64, s	25.1, CH_3_
11′	3.82, s	52.4, CH_3_

**Table 4:** Antiproliferative effects of centrapalus coumarins N (**2**) and O (**5**) on human gynecological cancer cell lines and NIH/3T3 mouse fibroblasts.

Cell Line	Conc. (µM)	Inhibition (%) ± SEM (Calculated IC_50_ Value, µM)		
		2	5	Cisplatin
MCF7	10	34.64 ± 2.19	– *	66.91 ± 1.81
	30	44.18 ± 1.76	35.50 ± 1.51	96.80 ± 0.35 (5.78)
MDA-MB-231	10	–	–	42.72 ± 2.68
	30	25.38 ± 2.06	25.53 ± 1.20	86.44 ± 0.42 (10.17)
HeLa	10	–	–	42.61 ± 2.33
	30	41.79 ± 1.42	31.31 ± 1.18	99.93 ± 0.26 (12.43)
SiHa	10	–	–	60.98 ± 0.92
	30	30.72 ± 1.49	33.34 ± 1.32	88.95 ± 0.53 (4.29)
A2780	10	27.72 ± 1.34	27.41 ± 1.55	83.57 ± 2.21
	30	66.21 ± 0.70 (20.11)	69.28 ± 1.12 (15.99)	95.02 ± 0.28 (1.30)
NIH/3T3	10	–	–	73.88 ± 1.63
	30	48.54 ± 2.20	28.36 ± 2.50	97.10 ± 0.15 (5.50)

*: Inhibition less than 20%.

## Data Availability

The data presented in this study are available on request from the corresponding author (J.H.).

## Supplementary Materials

The following supporting information can be downloaded at: https://www.mdpi.com/article/10.3390/pharmaceutics16070907/s1, HRESIMS and 1D and 2D NMR spectra of isolated compounds **1**–**5**, energy minimalized 3D structures, single-crystal X-ray diffraction data for compound **5**, and isolation flow chart. References [27,28,29,30] are cited in the Supplementary Materials.
