# Supplementary material for: New Members of the Centrapalus Coumarin and Pauciflorin Series from Centrapalus pauciflorus
Source: Pharmaceutics. 2024 Jul 6;16(7):907. doi: 10.3390/pharmaceutics16070907 (PMC11279841; doi:10.3390/pharmaceutics16070907)
Supplement: Supplementary file 1 [file pharmaceutics-16-00907-s001.zip › pharmaceutics-3036660-supplementary.pdf]

SUPPORTING INFORMATION

New Members of the Centrapalus Coumarin and Pauciflorin Series from *Centrapalus pauciflorus*

Muhammad Bello Saidu 1, Gordana Krstić 2, Petra Bombicz 3, Sourav De 3,
Anita Barta 1, Hazhmat Ali 4, István Zupkó 4, Róbert Berkecz 5, Umar Shehu Gallah 6,
Dóra Rédei 1 and Judit Hohmann 1,7,*

1. Department of Pharmacognosy, University of Szeged, Eötvös u. 6, 6720 Szeged, Hungary
2. Faculty of Chemistry, University of Belgrade, Studentski trg 12-16, 11158 Belgrade, Serbia
3. Centre for Structural Science, HUN-REN Research Centre for Natural Sciences, Magyar Tudósok körútja 2, 1117 Budapest, Hungary
4. Institute of Pharmacodynamics and Biopharmacy, University of Szeged, Eötvös u. 6, 6720 Szeged, Hungary
5. Institute of Pharmaceutical Analysis, University of Szeged, Somogyi u. 4, 6720 Szeged, Hungary
6. Bioresource Department, National Research Institute for Chemical Technology (NARICT), PMB 1052 Zaria, Nigeria
7. HUN-REN–USZ Biologically Active Natural Products Research Group, University of Szeged, Eötvös u. 6, 6720 Szeged, Hungary

* Correspondence: hohmann.judit@szte.hu

CONTENT

I. DATA IN CONNECTION TO STRUCTURE DETERMINATION

Figure S1. Energy minimalized 3D structure of **2** with key atom distances 4
Figure S2. Energy minimalized 3D structure of **3** with key atom distances 4
Figure S3. ^{1}H-NMR spectrum and signal assignments of compound **1** ($CDCl_3$, 500 MHz) 5
Figure S4. ^{13}C NMR JMOD spectrum and signal assignments of compound **1** ($CDCl_3$, 125 MHz) .. 5
Figure S5. ^{1}H-^{1}H COSY spectrum of compound **1** ($CDCl_3$, 500 MHz)... 6
Figure S6. HSQC spectrum of compound **1** ($CDCl_3$, 500/125 MHz) 6
Figure S7A. HMBC spectrum of compound **1** ($CDCl_3$, 500/125 MHz) 7
Figure S7B. HMBC spectrum of compound **1** ($CDCl_3$, 600/150 MHz) 7
Figure S8. NOESY spectrum of compound **1** ($CDCl_3$, 500 MHz) 8
Figure S9. HRESIMS spectrum of compound **1**... 8
Figure S10. ^{1}H-NMR spectrum of compound **2** ($CDCl_3$, 500 MHz) 9
Figure S11. ^{13}C NMR JMOD spectrum of compound **2** ($CDCl_3$, 125 MHz) 9
Figure S12. ^{1}H-^{1}H COSY spectrum of compound **2** ($CDCl_3$, 500 MHz)... 10
Figure S13. HSQC spectrum of compound **2** ($CDCl_3$, 500/125 MHz) 10
Figure S14. HMBC spectrum of compound **2** ($CDCl_3$, 500/125 MHz) 11
Figure S15. NOESY spectrum of compound **2** ($CDCl_3$, 500 MHz) 11
Figure S16. HRESIMS spectrum of compound **2**.. 12
Figure S17. ^{1}H-NMR spectrum of compound **3** ($CDCl_3$, 500 MHz).. 12
Figure S18. ^{13}C NMR JMOD spectrum of compound **3** ($CDCl_3$, 125 MHz) 13
Figure S19. ^{1}H-^{1}H COSY spectrum of compound **3** ($CDCl_3$, 500 MHz)... 13
Figure S20. HSQC spectrum of compound **3** ($CDCl_3$, 500/125 MHz) 14
Figure S21. HMBC spectrum of compound **3** ($CDCl_3$, 500/125 MHz) 14
Figure S22. NOESY spectrum of compound **3** ($CDCl_3$, 500 MHz) 15
Figure S23. HRESIMS spectrum of compound **3**.. 15
Figure S24. ^{1}H-NMR spectrum of compound **4** ($CDCl_3$, 500 MHz).. 16
Figure S25. ^{13}C NMR JMOD spectrum of compound **4** ($CDCl_3$, 125 MHz) 16
Figure S26. ^{1}H-^{1}H COSY spectrum of compound **4** ($CDCl_3$, 500 MHz)... 17
Figure S27. HSQC spectrum of compound **4** ($CDCl_3$, 500/125 MHz) 17
Figure S28. HMBC spectrum of compound **4** ($CDCl_3$, 500/125 MHz) 18
Figure S29. NOESY spectrum of compound **4** ($CDCl_3$, 500 MHz) 18
Figure S30. HRESIMS spectrum of compound **4**.. 19
Figure S31. ^{1}H-NMR spectrum of compound **5** ($CDCl_3$, 500 MHz).. 19
Figure S32. ^{13}C NMR JMOD spectrum of compound **5** ($CDCl_3$, 125 MHz) 20
Figure S33. ^{1}H-^{1}H COSY spectrum of compound **5** ($CDCl_3$, 500 MHz)... 20
Figure S34. HSQC spectrum of compound **5** ($CDCl_3$, 500/125 MHz) 21
Figure S35. HMBC spectrum of compound **5** ($CDCl_3$, 500/125 MHz) 21
Figure S36. HRESIMS spectrum of compound **5**.. 22

II. STRUCTURE DETERMINATION OF COMPOUND 1 BY SINGLE CRYSTAL X-RAY DIFFRACTION 23

Scheme S1. Formula of (*E)*-methyl 3-(4,10-dimethyl-5-oxo-4-vinyl-2,3,4,5-tetrahydropyrano[3,2-c]chromen-2-yl)-2-methylacrylate **5** (VP83) 23

Scheme S2. The measured transparent, block type single crystal of **5** 23

Figure S37. Molecular structure diagram of compound **5** showing atom labelling. 24

Figure S38. Molecular structure diagrams of compound **5**. 25

Figure S39. Packing diagram of **5**. 25

Figure S40. Intra- and intermolecular interactions in the crystal structure of compound **5** 26

Figure S41. The calculated BFDH morphology of the crystal of **5** 27

Figure S42. The calculated powder diffraction pattern of **5** 27

Experimental 27

Table S1. Crystal data and details of the structure determination and refinement of **5** 28

Table S2. Atomic coordinates and equivalent isotropic displacement parameters of **5** 29

Table S3. Hydrogen coordinates and isotropic displacement parameters of **5**. 30

Table S4. Anisotropic displacement parameters of **1**. 31

Table S5. Bond lengths [Å] of **5**. 32

Table S6. Bond angles [°] of **5** 33

Table S7. Torsion angles [°] of **5**. 34

Table S8. Intra- and intermolecular interactions in **5**. 35

III. References 35

IV. Figure S43. Isolation flow chart 36

I. DATA IN CONNECTION TO STRUCTURE DETERMINATION

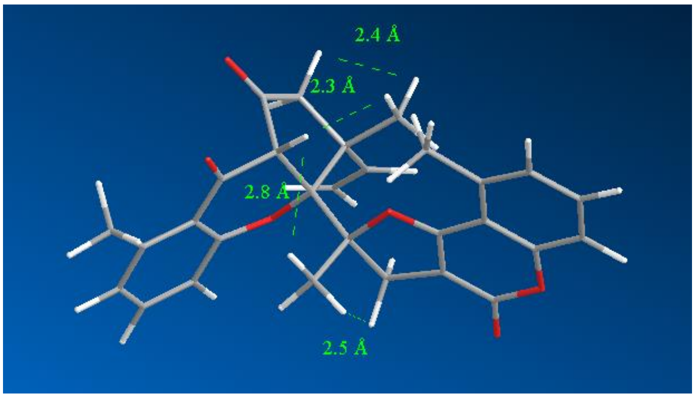

Figure S1. Energy minimalized 3D structure of **2** with key atom distances (H-3/H_3-10” 2.3 Å, H-3/H_3-9” 2.8 Å, H_3-10”/H-4”*β* 2.4 Å, H_3-9”/H-8”*β* 2.5 Å).

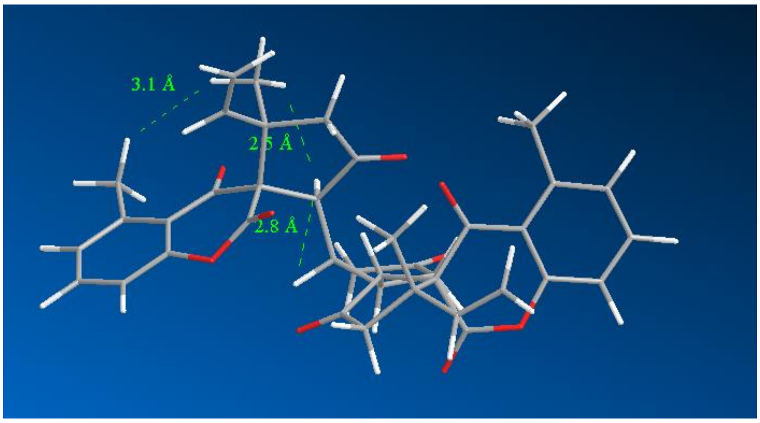

Figure S2. Energy minimalized 3D structure of **3** with key atom distances (H_3-9/H_3-10’ 3.1 Å, H_3-10’/H-6’ 2.5 Å, H-6’/H-7’ 2.8 Å).

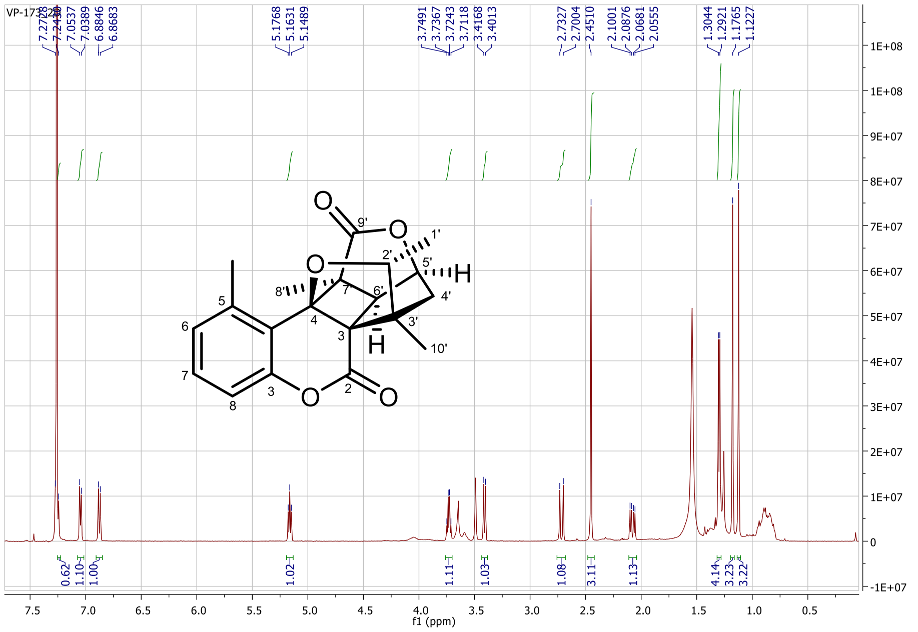

Position	1
2	-
3	-
4	-
4a	-
5	-
6	7.04, d (7.6)
7	7.26, t (7.6)
8	6.87, d (7.6)
8a	-
9	2.46, s
1′	1.30, d (6.3)
2′	3.73, q (6.3)
3′	-
4′α	2.72, d (16.2)
4′β	.08, dd (16.2. 7.0)
5′	5.16, t (7.0)
6′	3.40, d (7.0)
7′	-
8′	1.13, s
9′	-
10′	1.18, s
11′	-

Figure S3. ^{1}H-NMR spectrum and signal assignments of compound **1** ($CDCl_3$, 500 MHz)

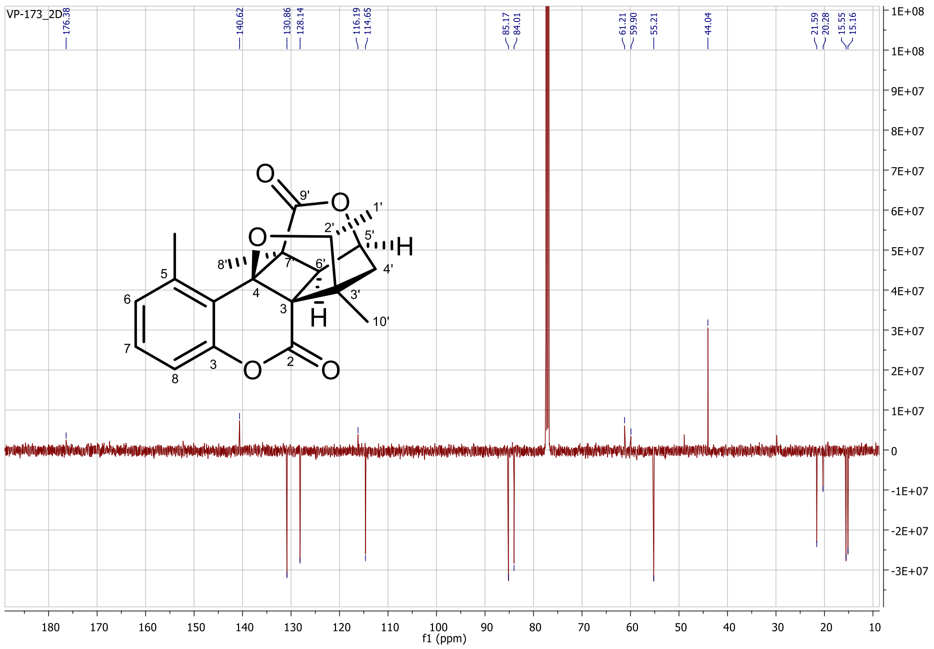

Position	1
2	167.5, C
3	59.9, C
4	84.7, C
4a	116.2, C
5	140.6, C
6	128.1, CH
7	130.8, CH
8	114.6, CH
8a	152.3, C
9	20.3, CH_3
1′	15.1, CH_3
2′	85.2, CH
3′	61.2, C
4′α	44.0, CH_2
4′β	
5′	84.0, CH
6′	55.2, CH
7′	48.9, C
8′	15.5, CH_3
9′	176.4, C
10′	21.6, CH_3
11′	-

Figure S4. ^{13}C NMR JMOD spectrum and signal assignments of compound **1** ($CDCl_3$, 125 MHz)

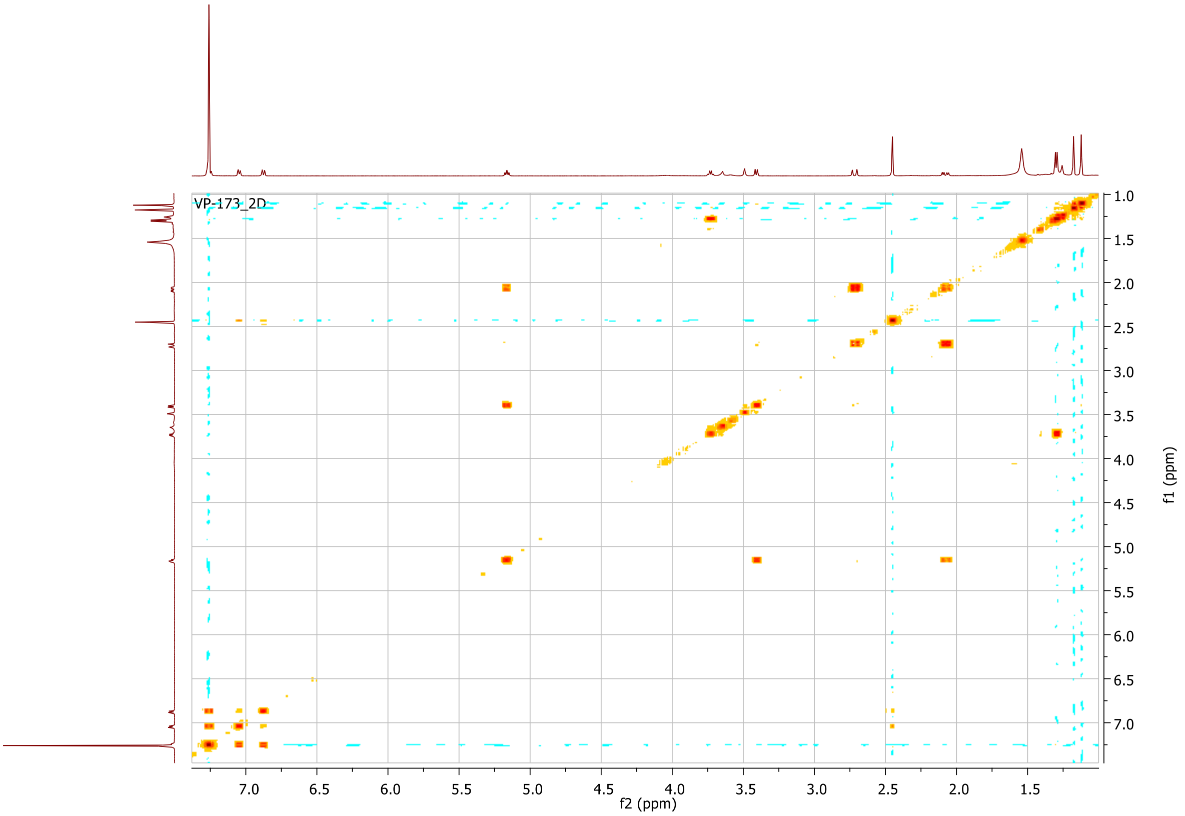

Figure S5. ^{1}H-^{1}H COSY spectrum of compound **1** ($CDCl_3$, 500 MHz)

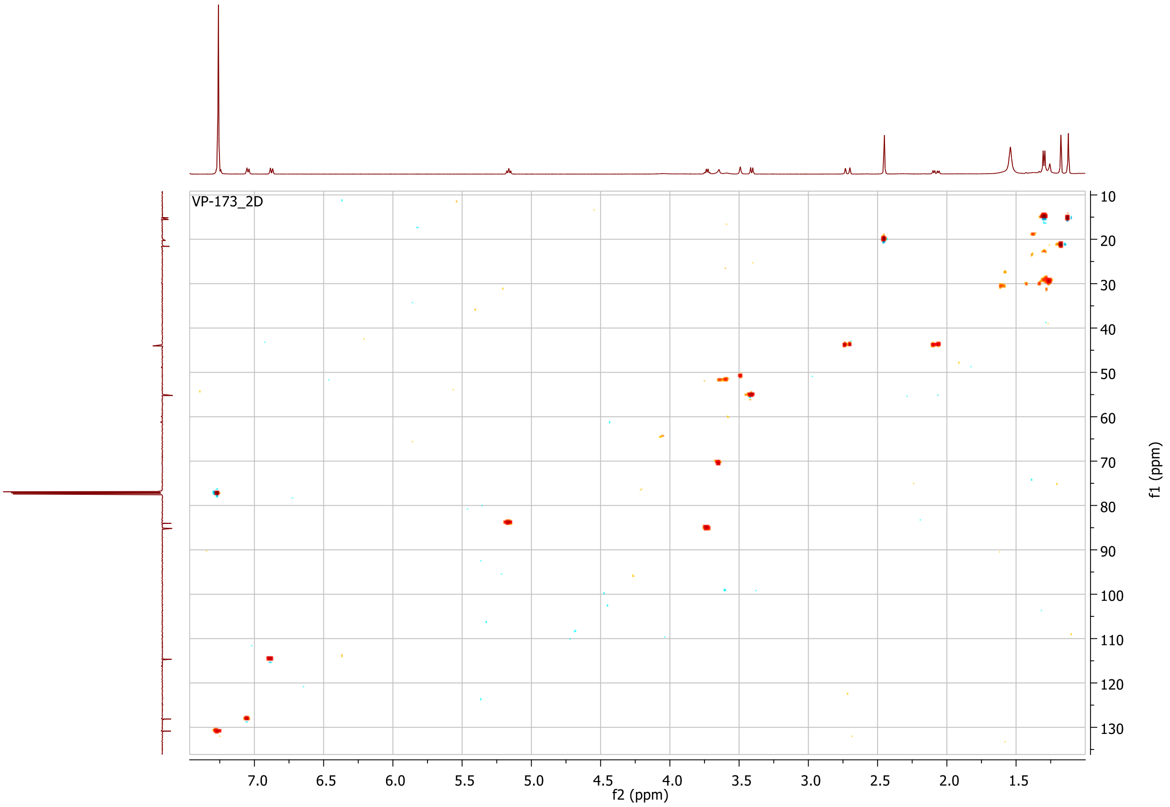

Figure S6. HSQC spectrum of compound **1** ($CDCl_3$, 500/125 MHz)

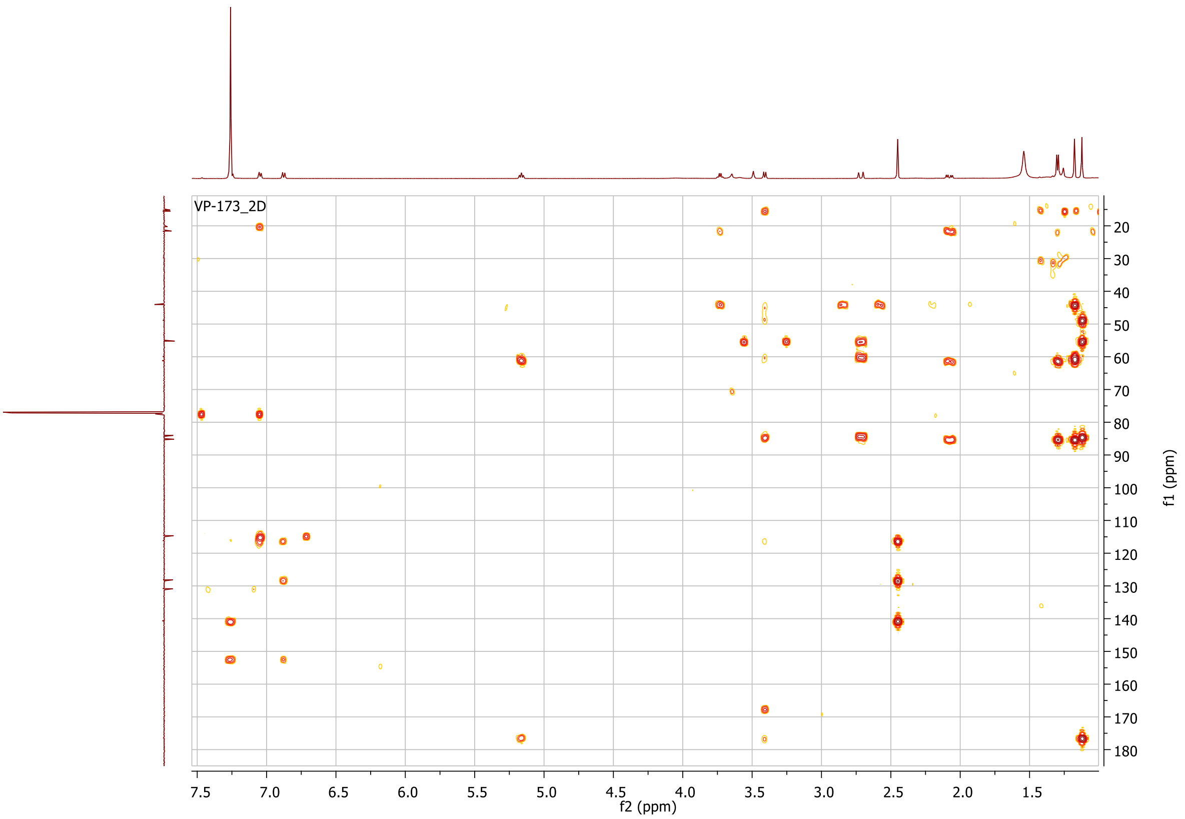

Figure S7A. HMBC spectrum of compound **1** ($CDCl_3$, 500/125 MHz)

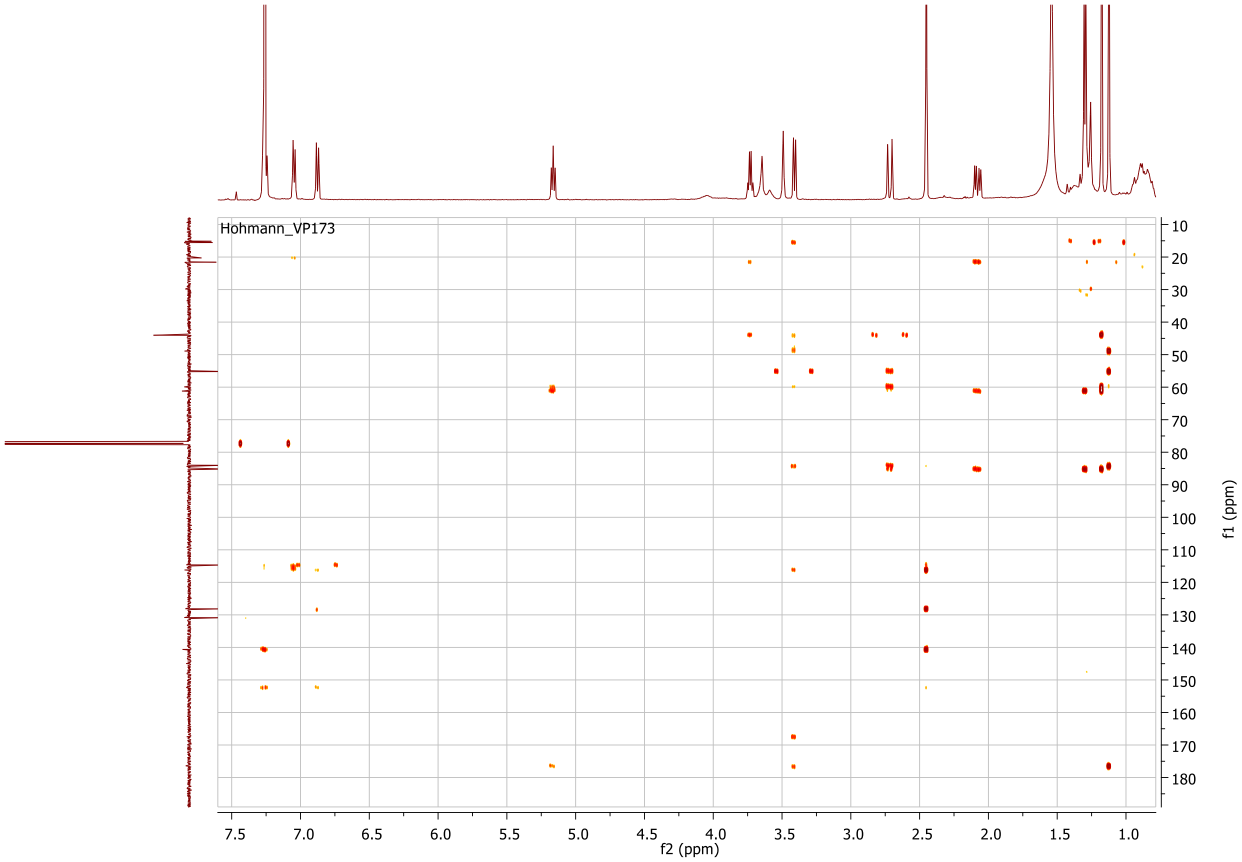

Figure S7B. HMBC spectrum of compound **1** ($CDCl_3$, 600/150 MHz)

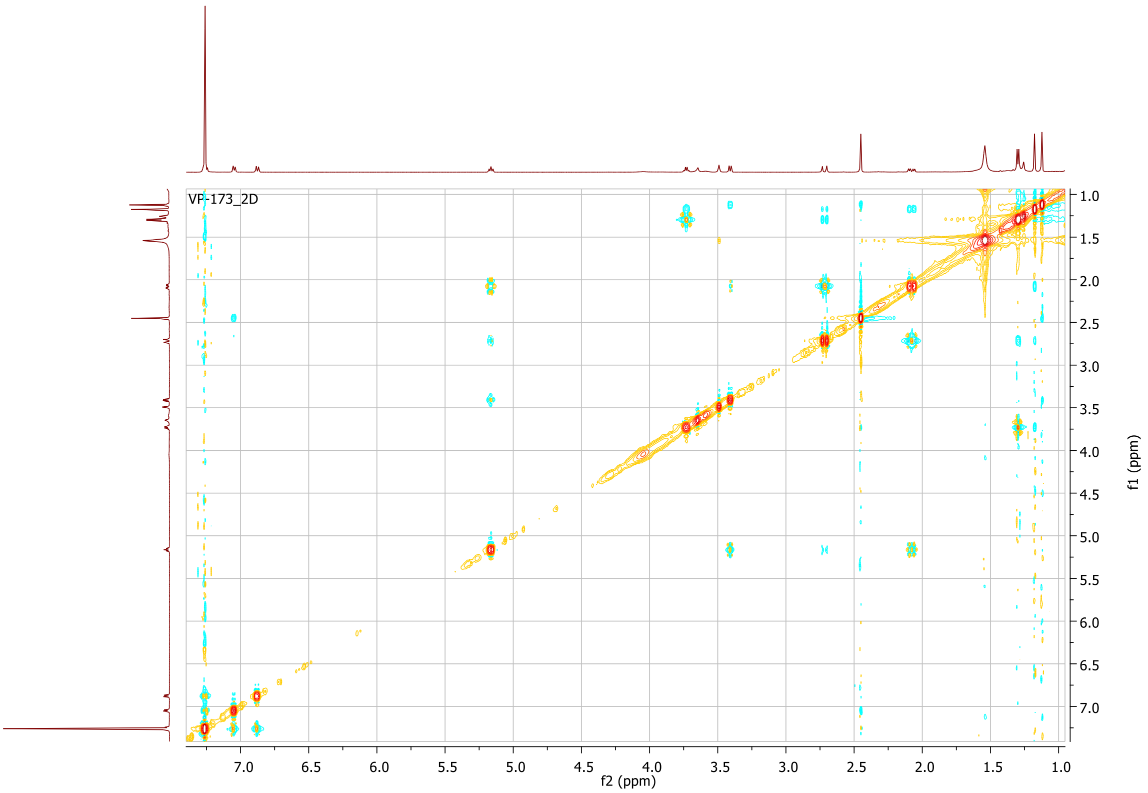

Figure S8. NOESY spectrum of compound **1** ($CDCl_3$, 500 MHz)

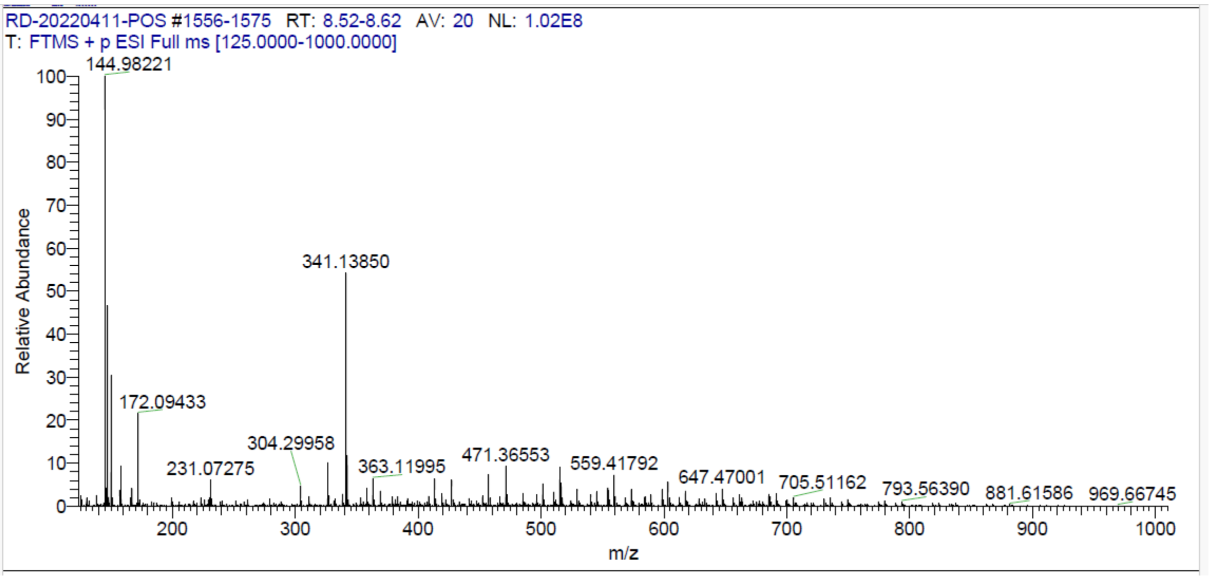

Figure S9. HRESIMS spectrum of compound **1**.

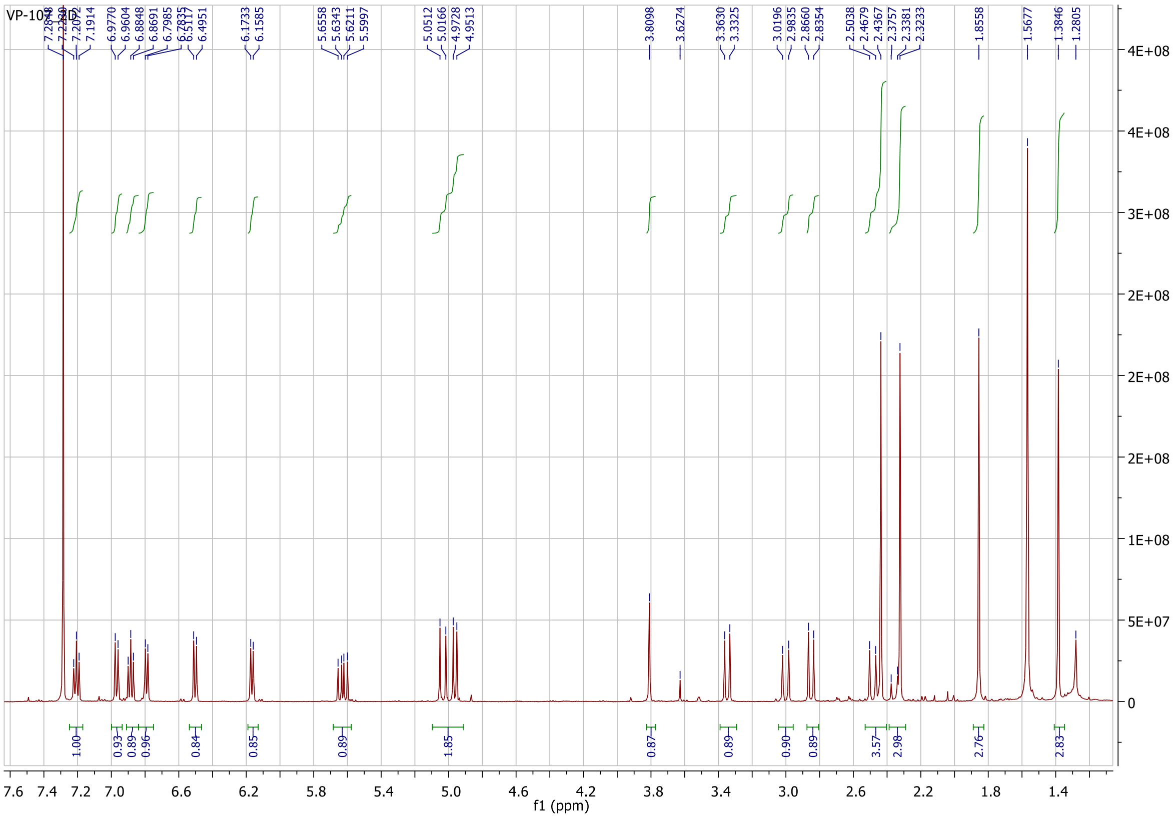

Figure S10. ^{1}H-NMR spectrum of compound **2** ($CDCl_3$, 500 MHz)

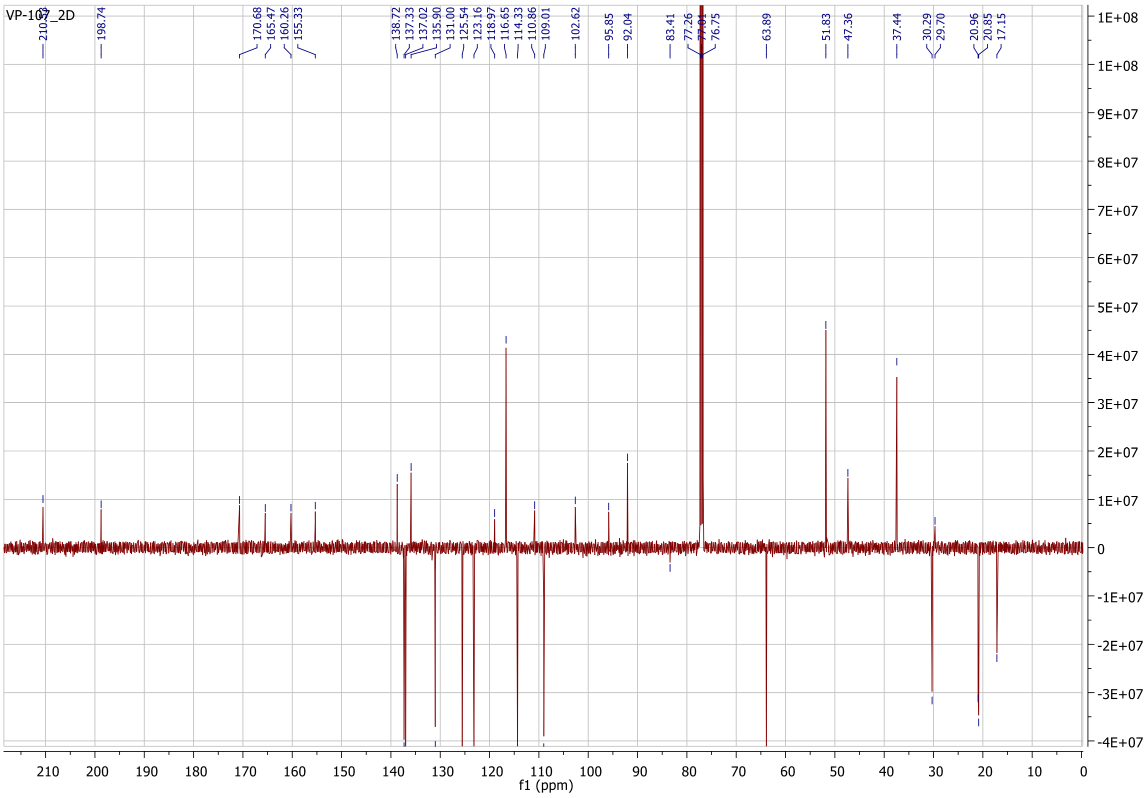

Figure S11. ^{13}C NMR JMOD spectrum of compound **2** ($CDCl_3$, 125 MHz)

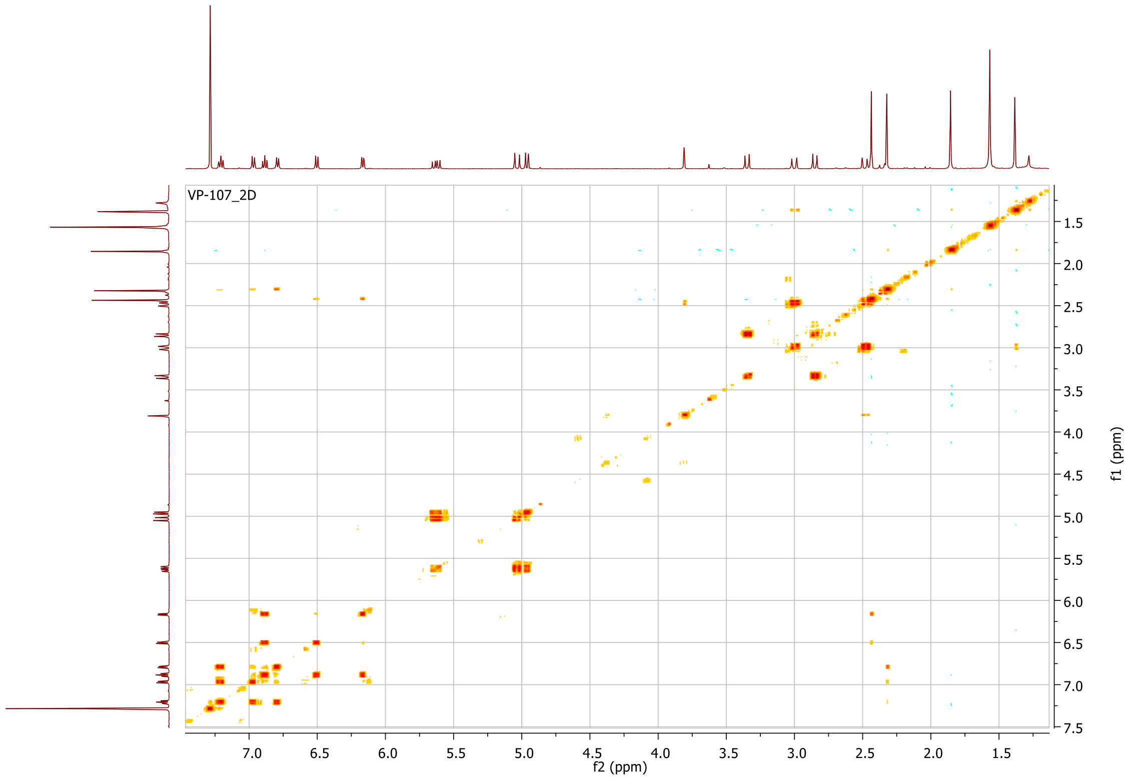

Figure S12. ^{1}H-^{1}H COSY spectrum of compound **2** ($CDCl_3$, 500 MHz)

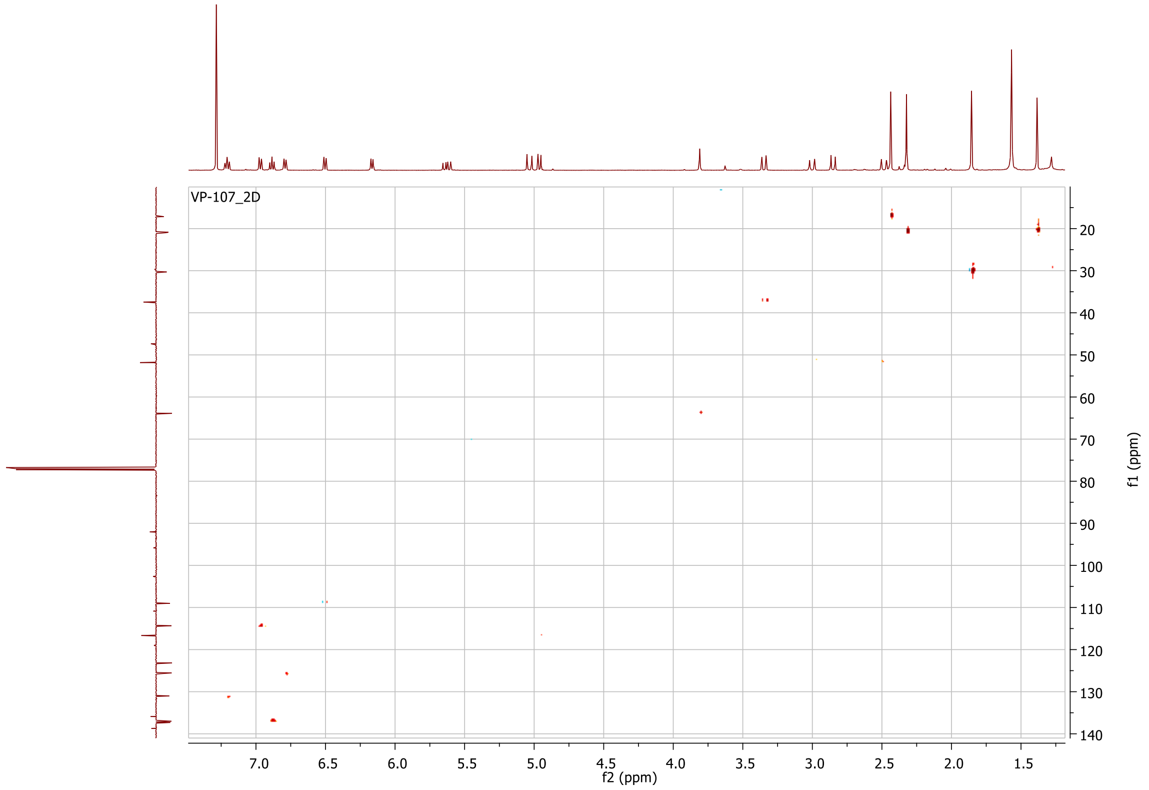

Figure S13. HSQC spectrum of compound **2** ($CDCl_3$, 500/125 MHz)

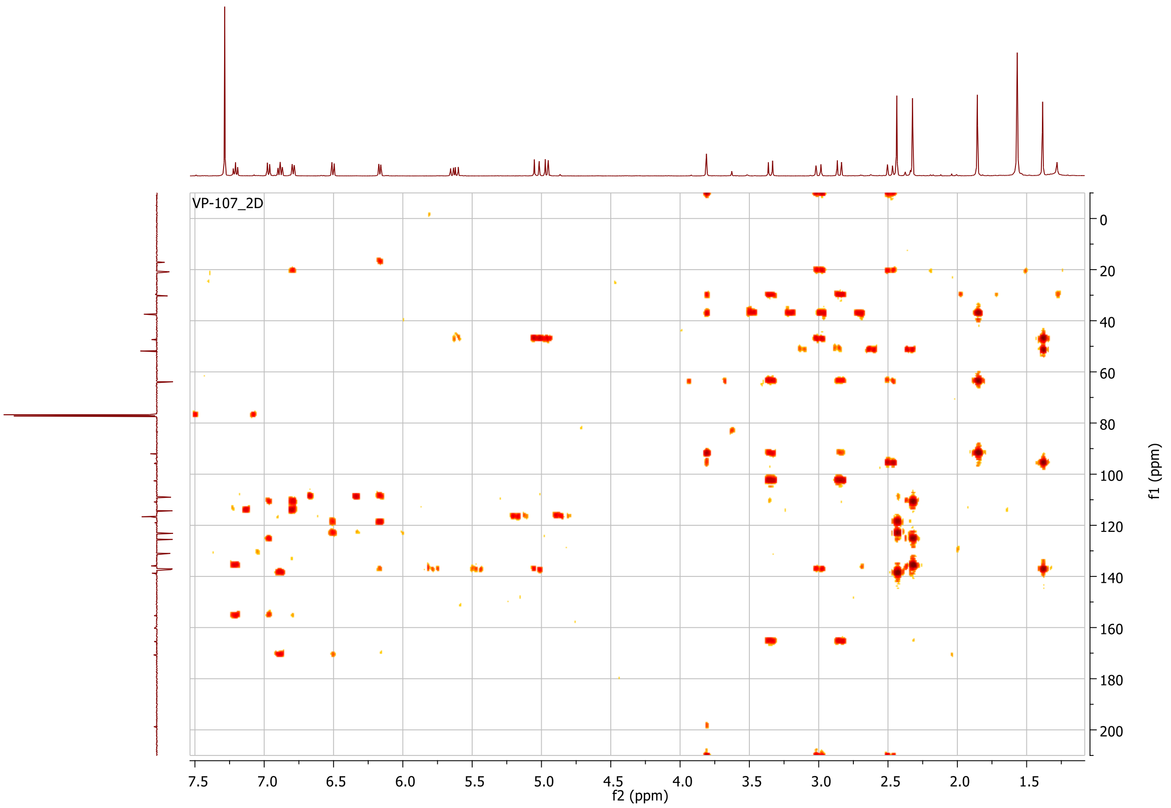

Figure S14. HMBC spectrum of compound **2** ($CDCl_3$, 500/125 MHz)

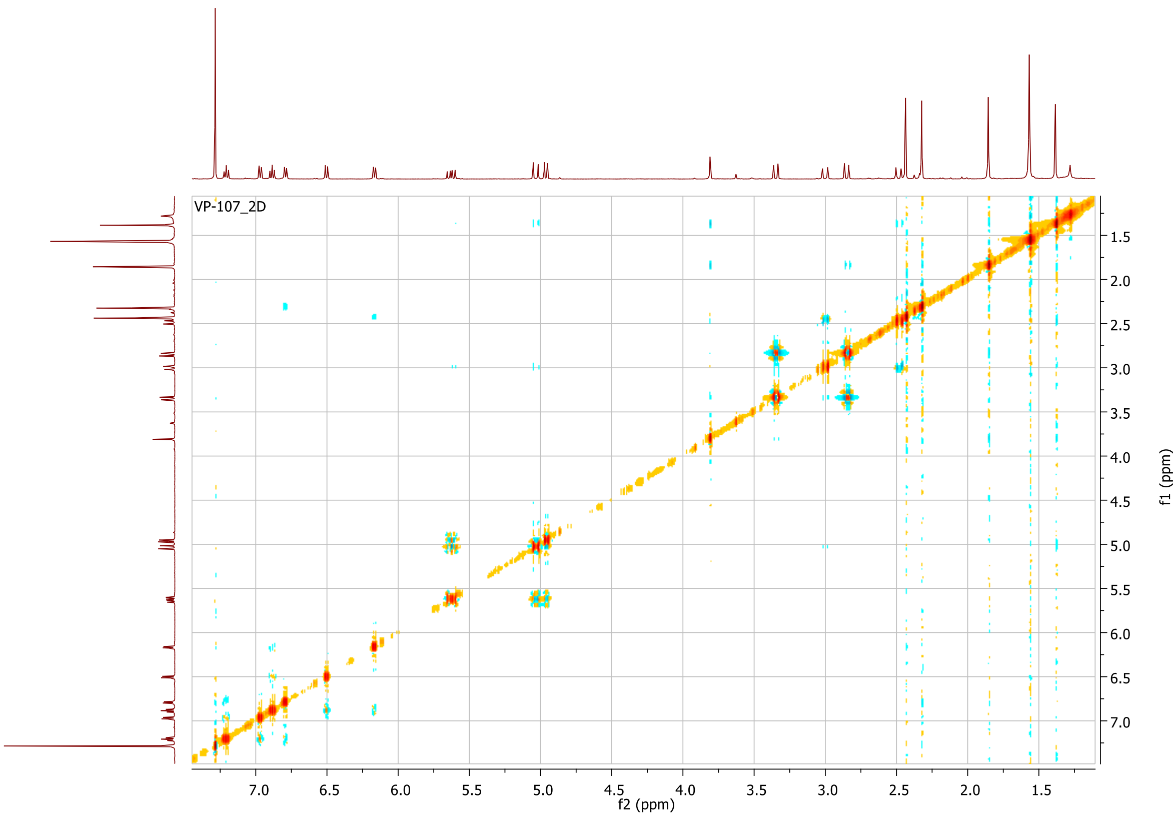

Figure S15. NOESY spectrum of compound **2** ($CDCl_3$, 500 MHz)

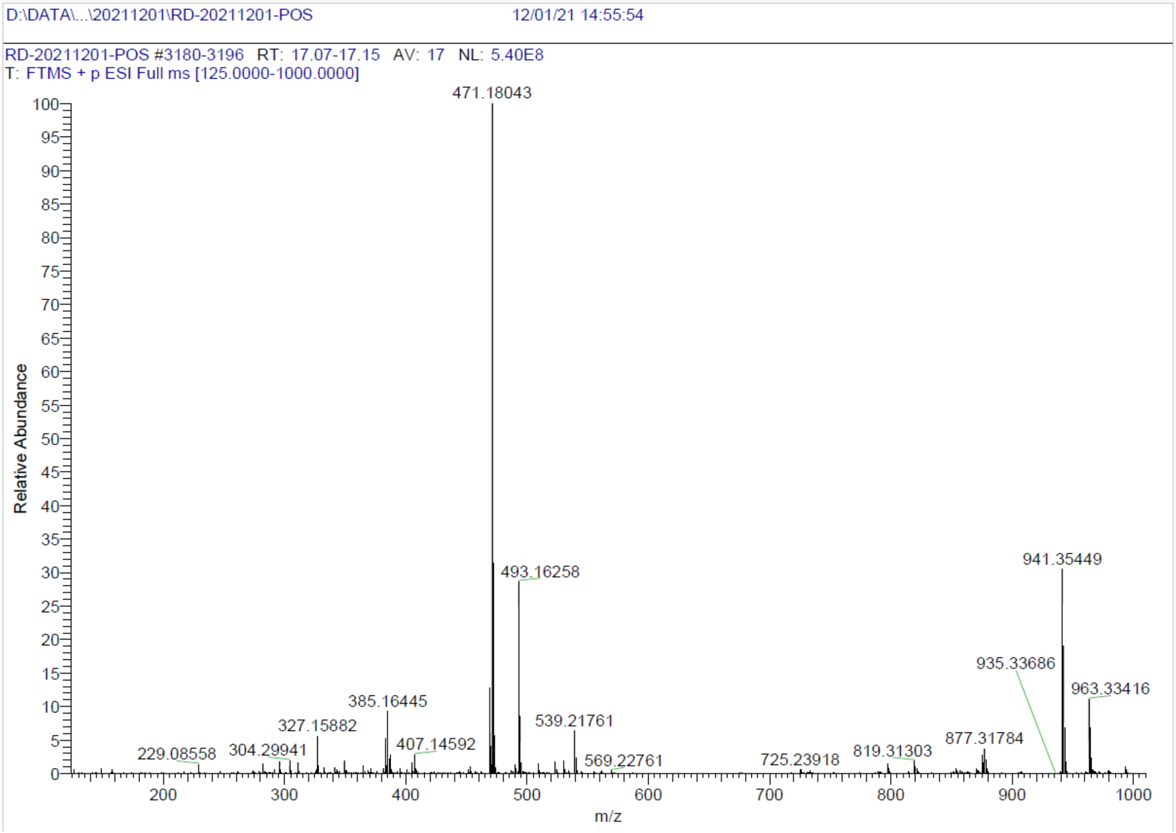

Figure S16. HRESIMS spectrum of compound **2**.

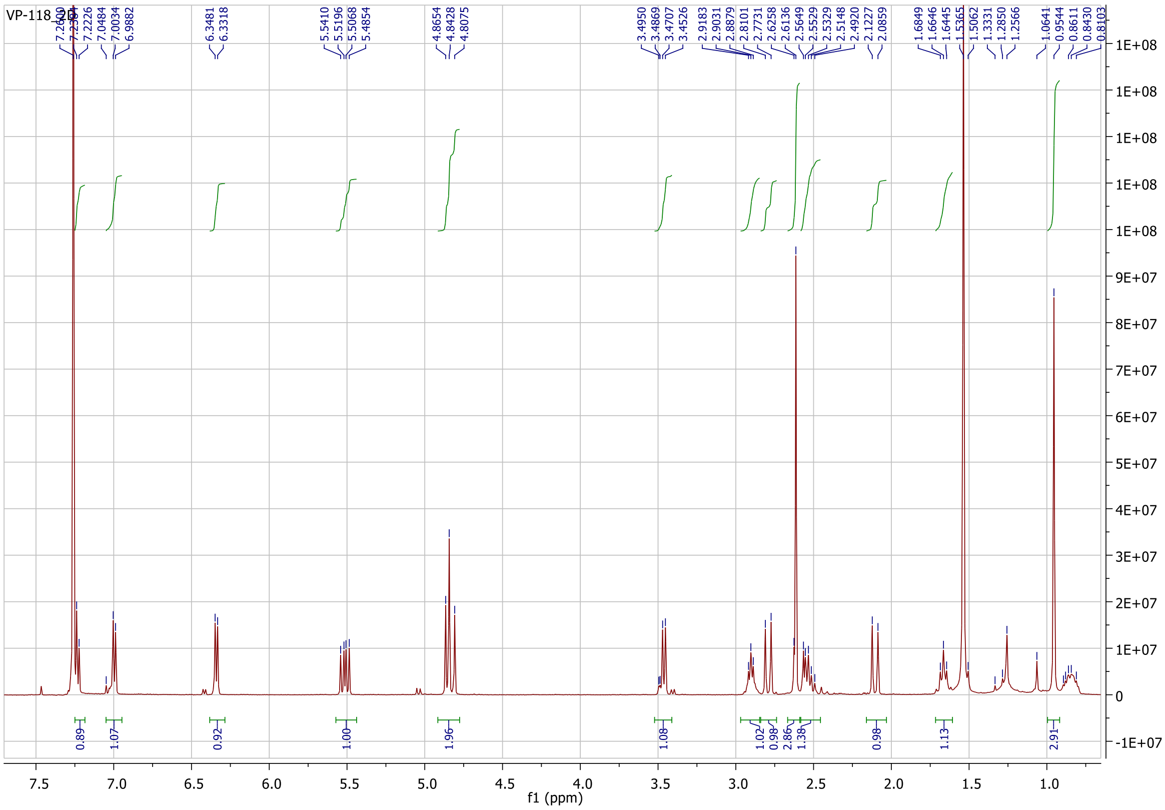

Figure S17. ^{1}H-NMR spectrum of compound **3** ($CDCl_3$, 500 MHz)

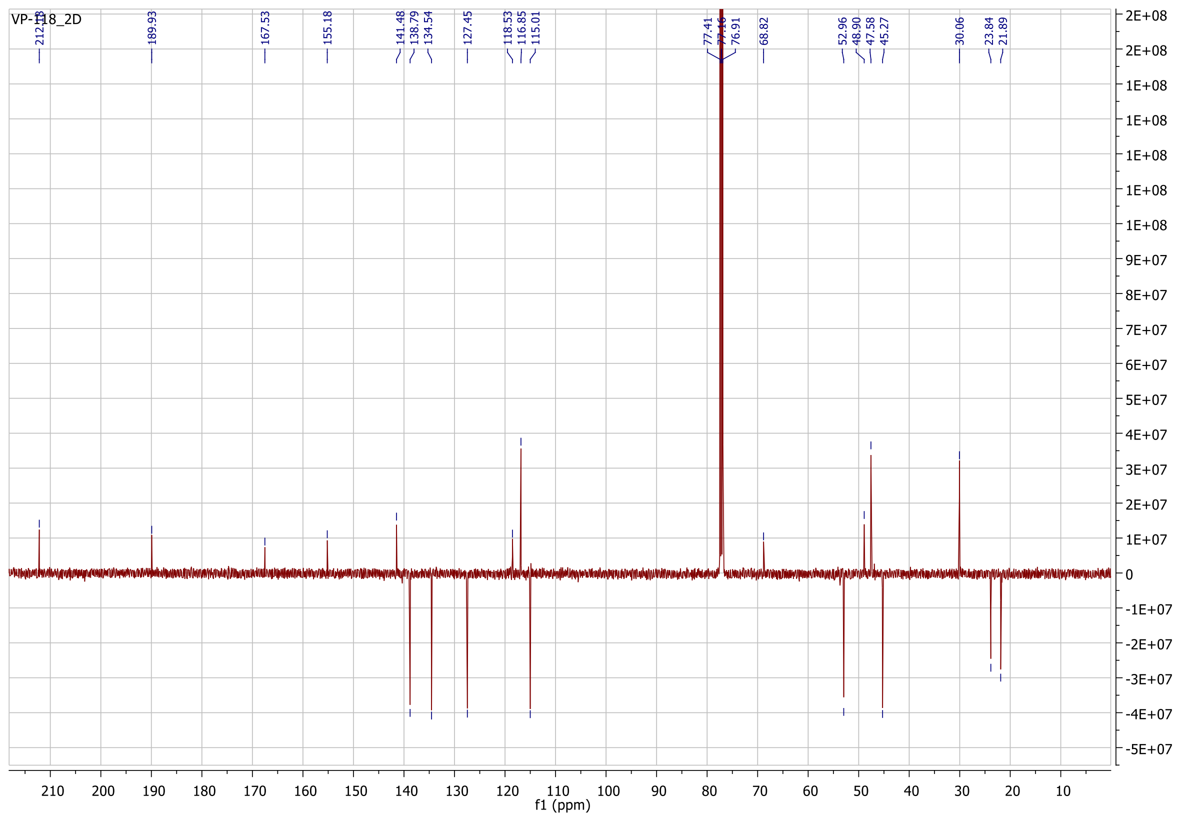

Figure S18. ^{13}C NMR JMOD spectrum of compound **3** ($CDCl_3$, 125 MHz)

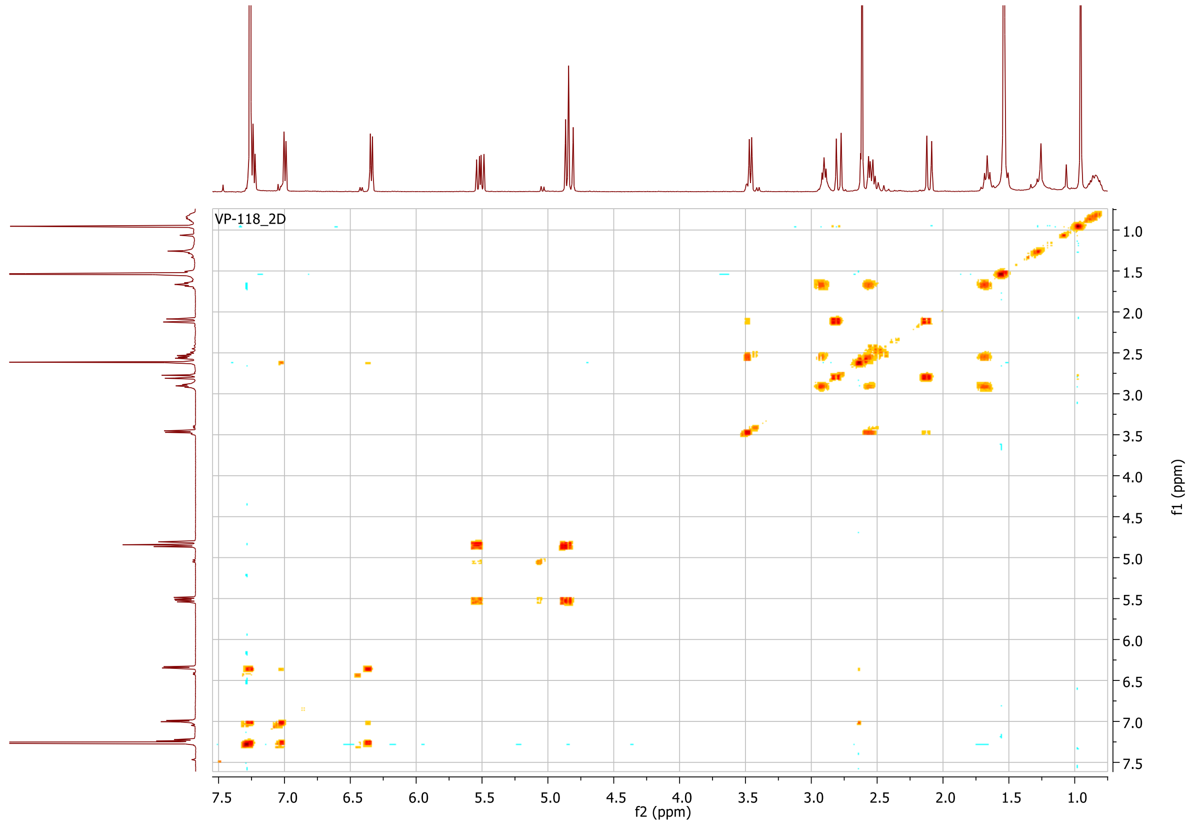

Figure S19. ^{1}H-^{1}H COSY spectrum of compound **3** ($CDCl_3$, 500 MHz)

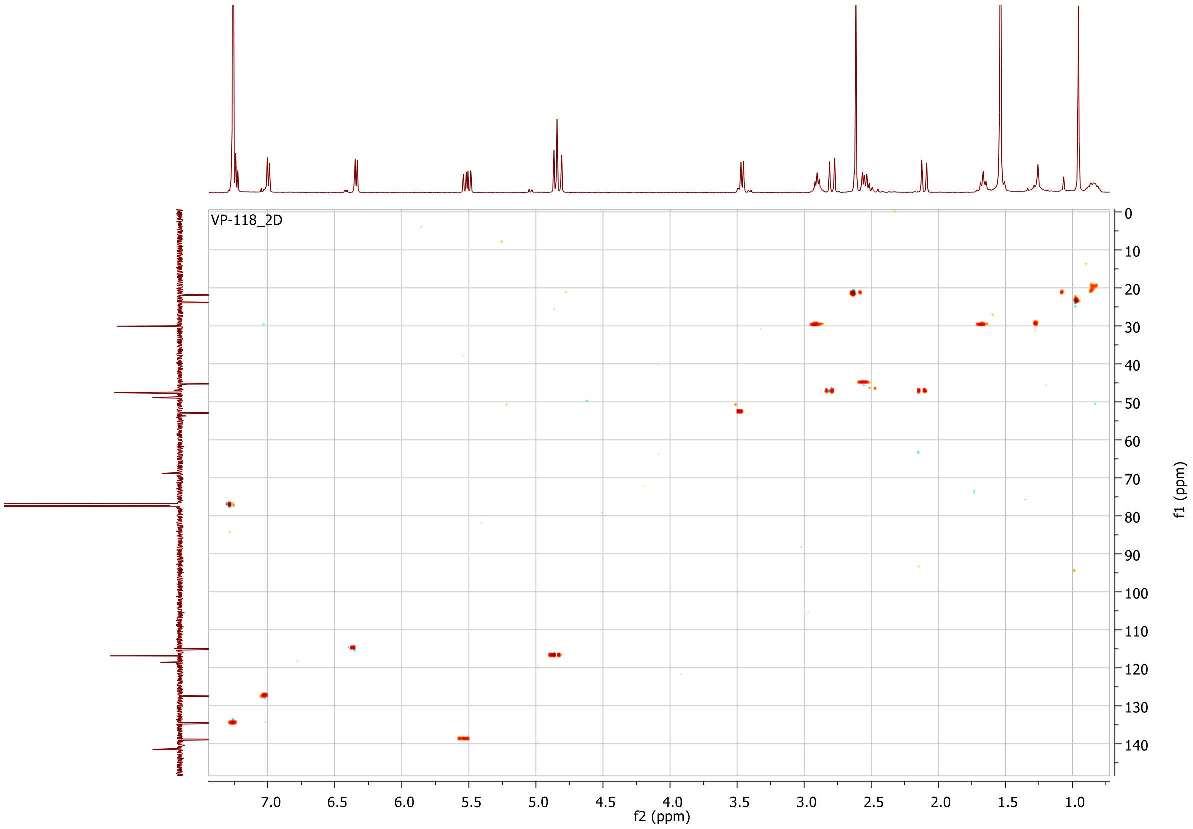

Figure S20. HSQC spectrum of compound **3** ($CDCl_3$, 500/125 MHz)

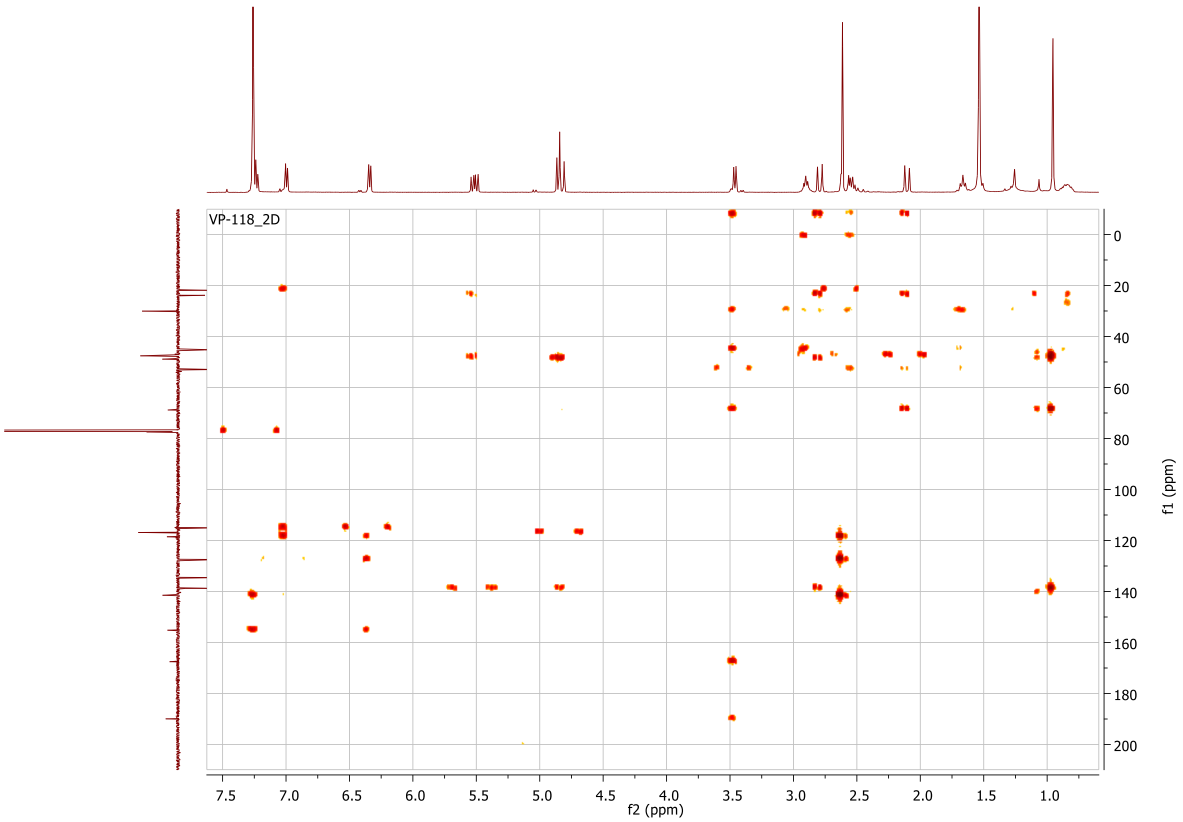

Figure S21. HMBC spectrum of compound **3** ($CDCl_3$, 500/125 MHz)

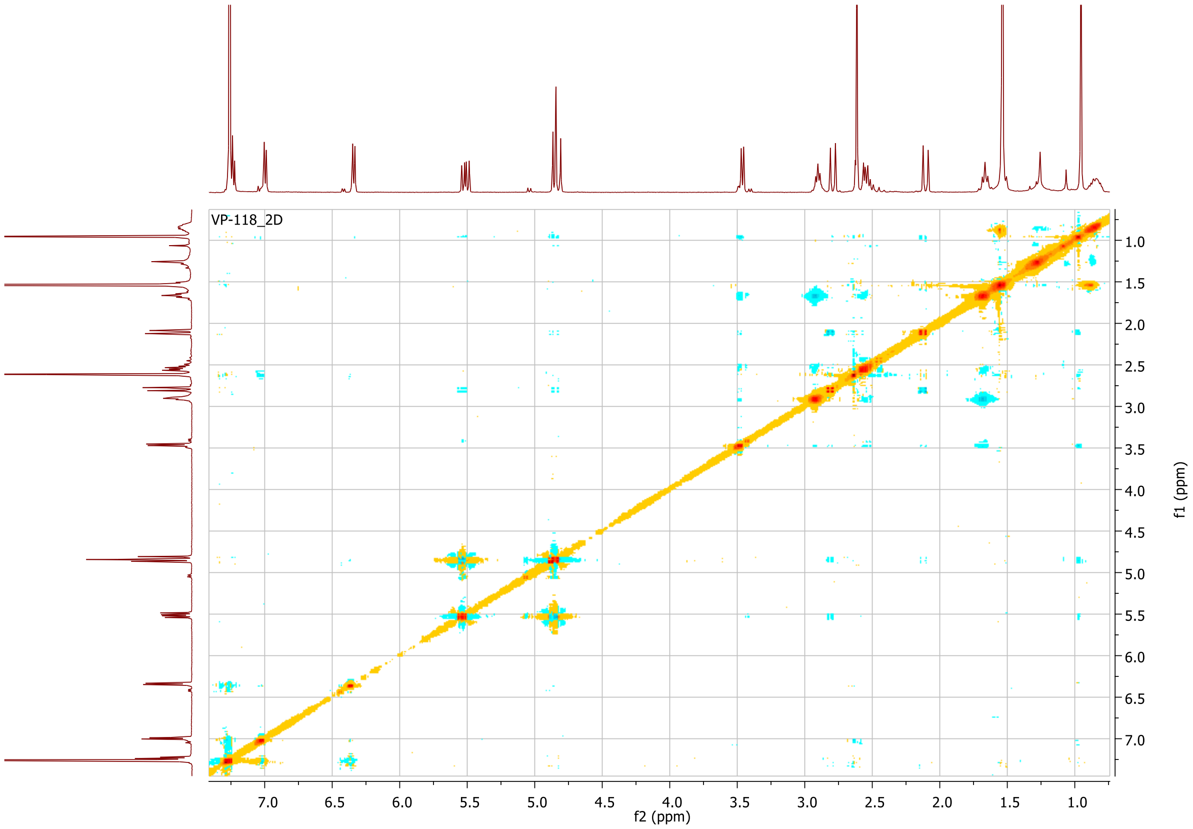

Figure S22. NOESY spectrum of compound **3** ($CDCl_3$, 500 MHz)

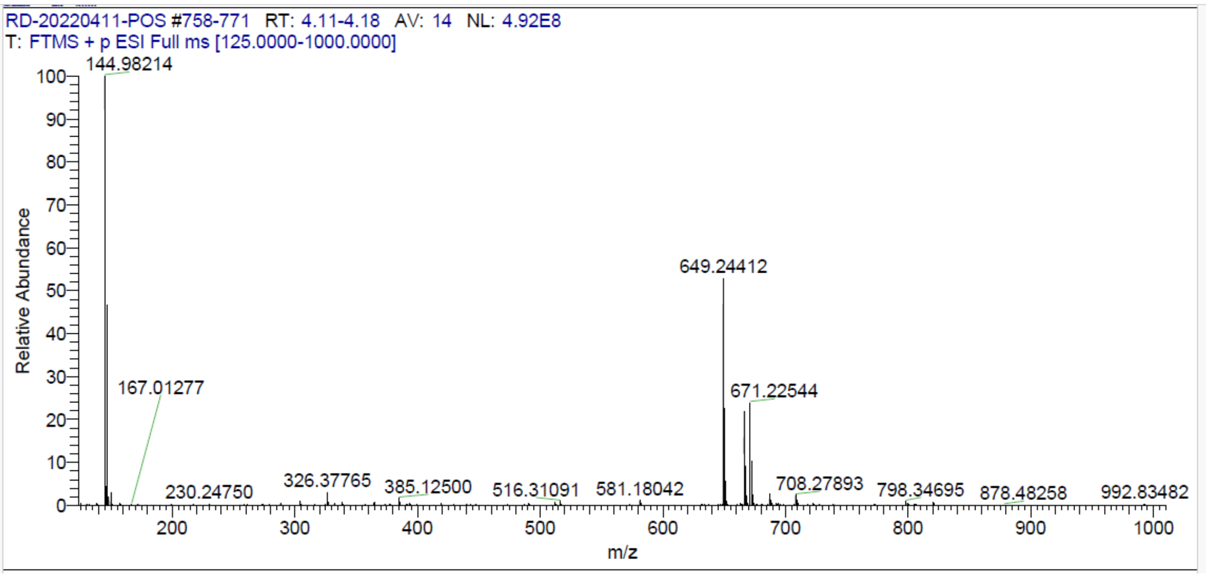

Figure S23. HRESIMS spectrum of compound **3**.

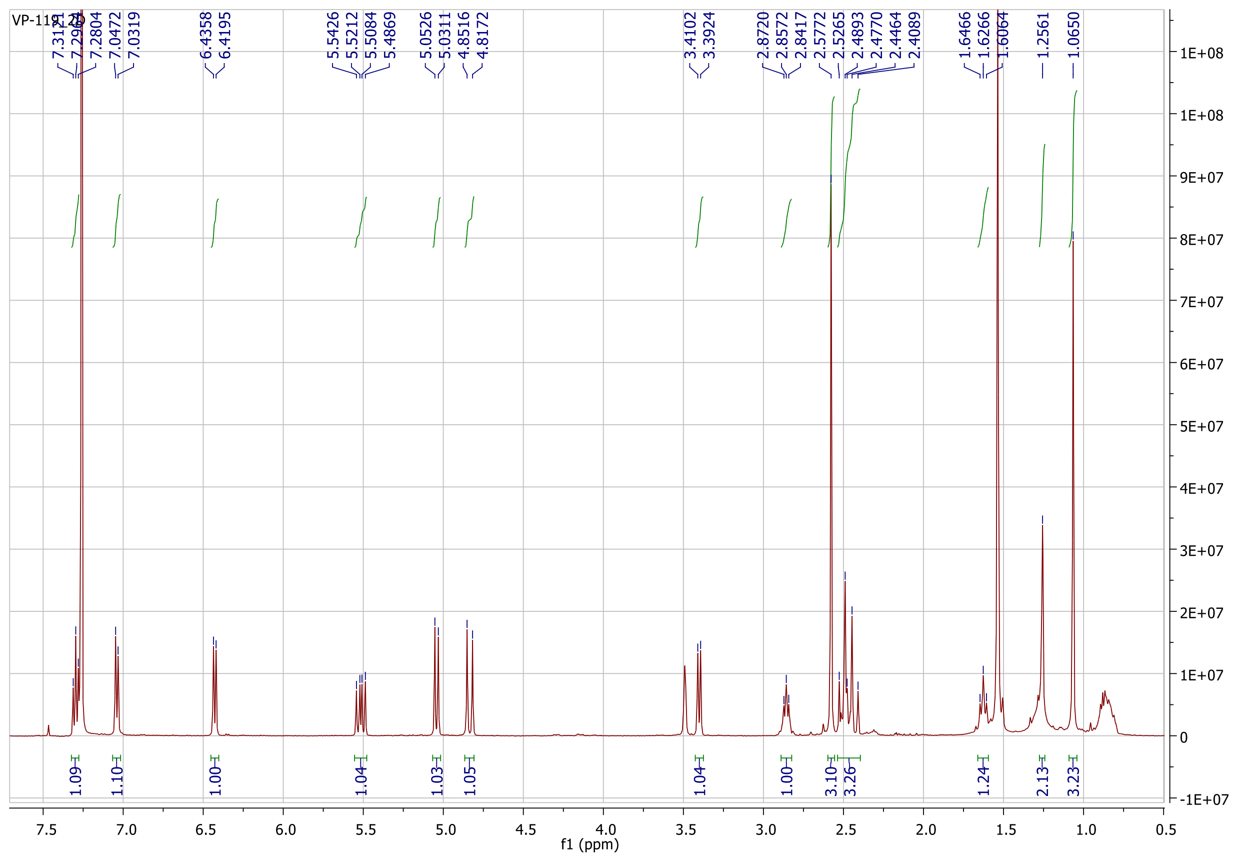

Figure S24. ^{1}H-NMR spectrum of compound **4** ($CDCl_3$, 500 MHz)

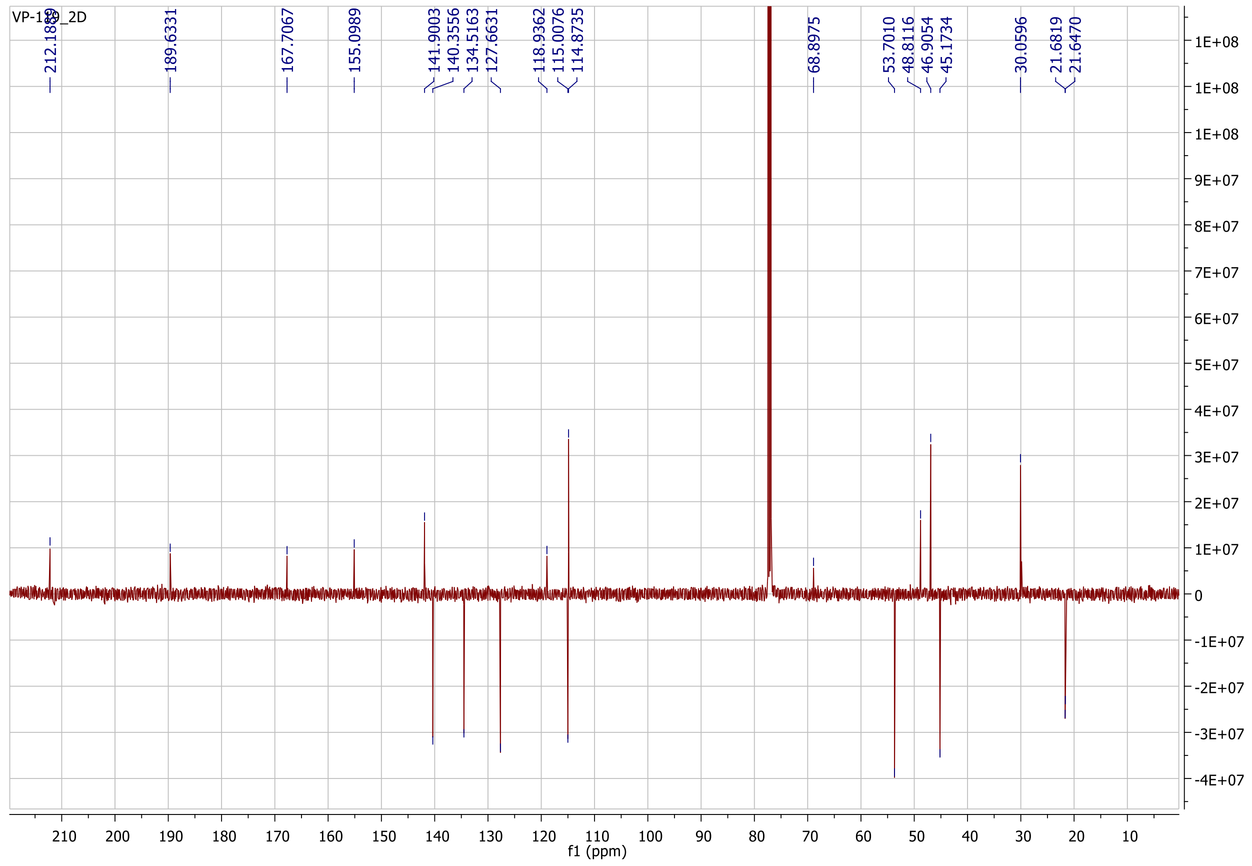

Figure S25. ^{13}C NMR JMOD spectrum of compound **4** ($CDCl_3$, 125 MHz)

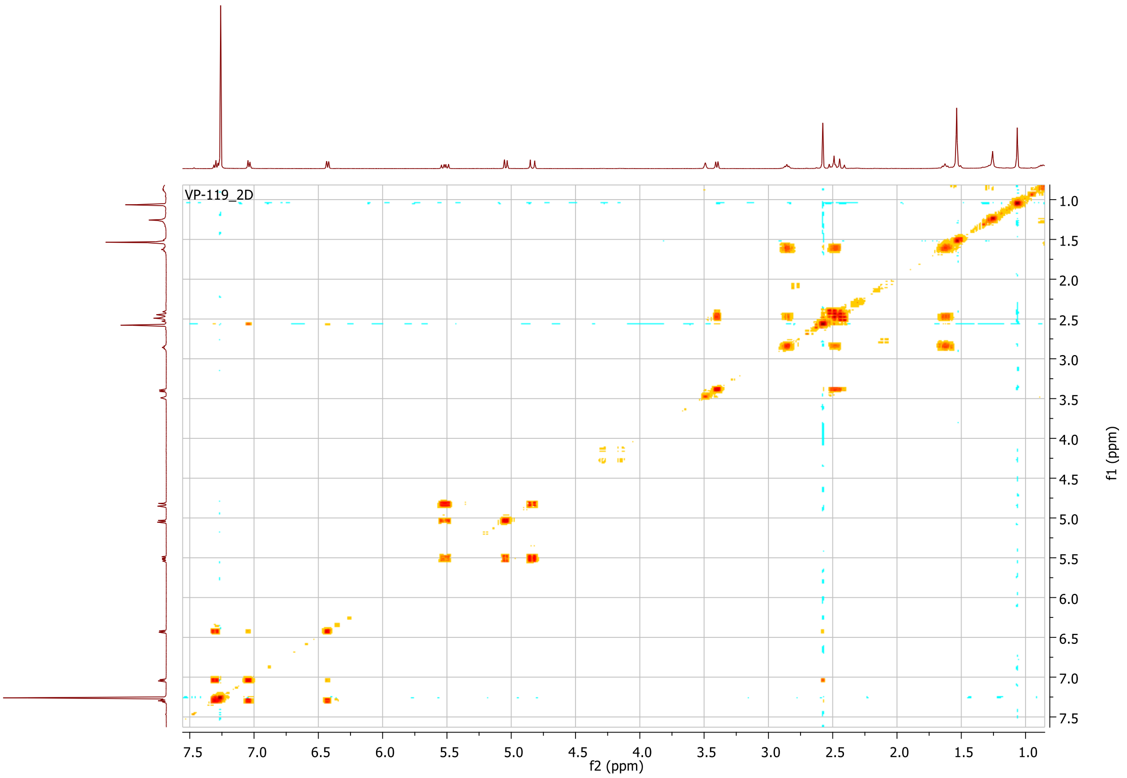

Figure S26. ^{1}H-^{1}H COSY spectrum of compound **4** ($CDCl_3$, 500 MHz)

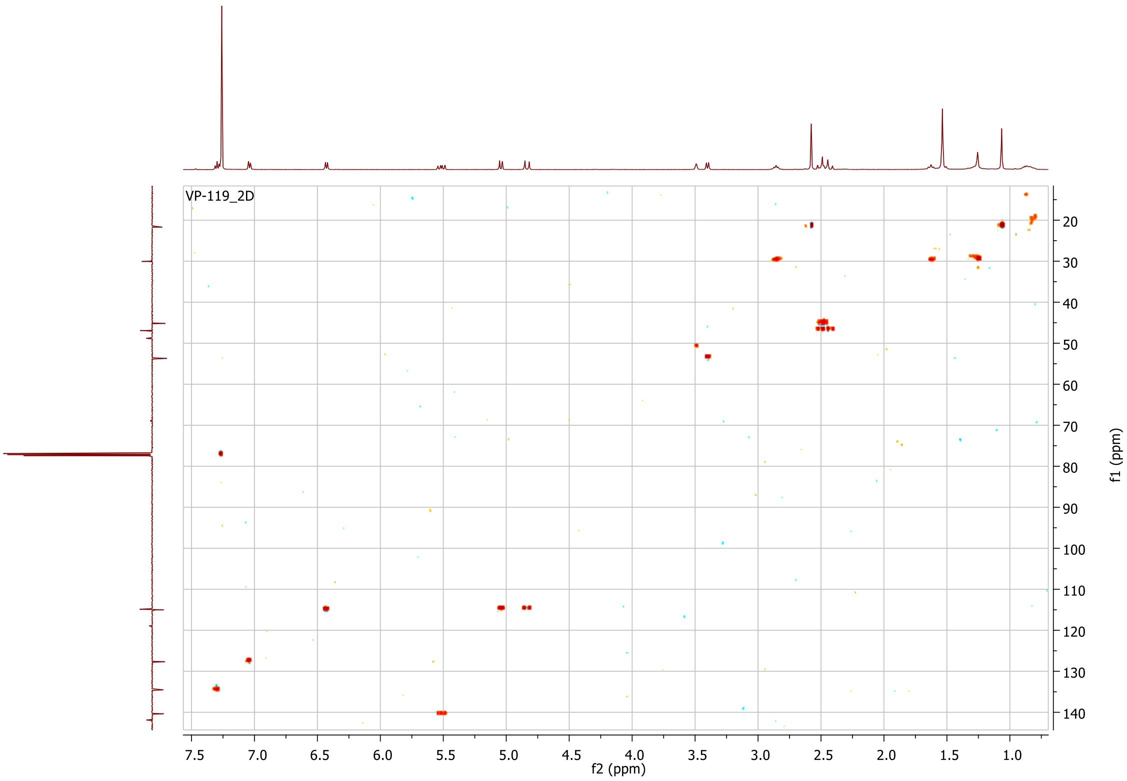

Figure S27. HSQC spectrum of compound **4** ($CDCl_3$, 500/125 MHz)

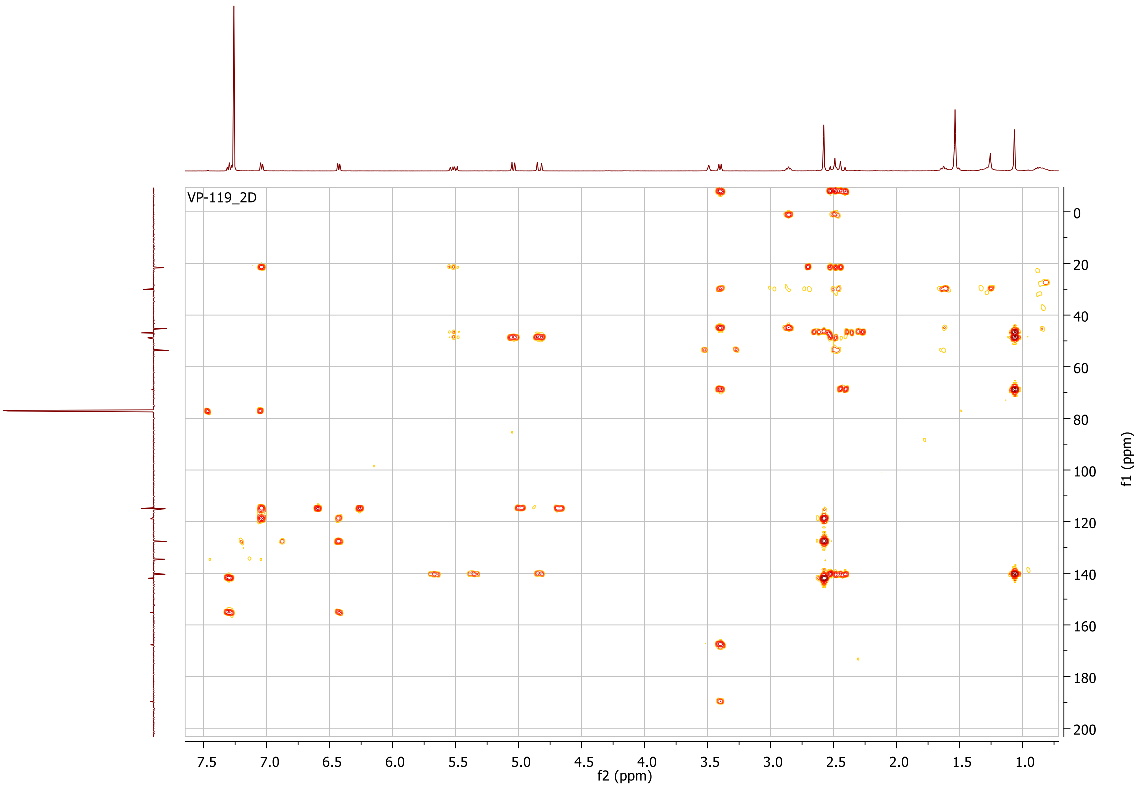

Figure S28. HMBC spectrum of compound **4** ($CDCl_3$, 500/125 MHz)

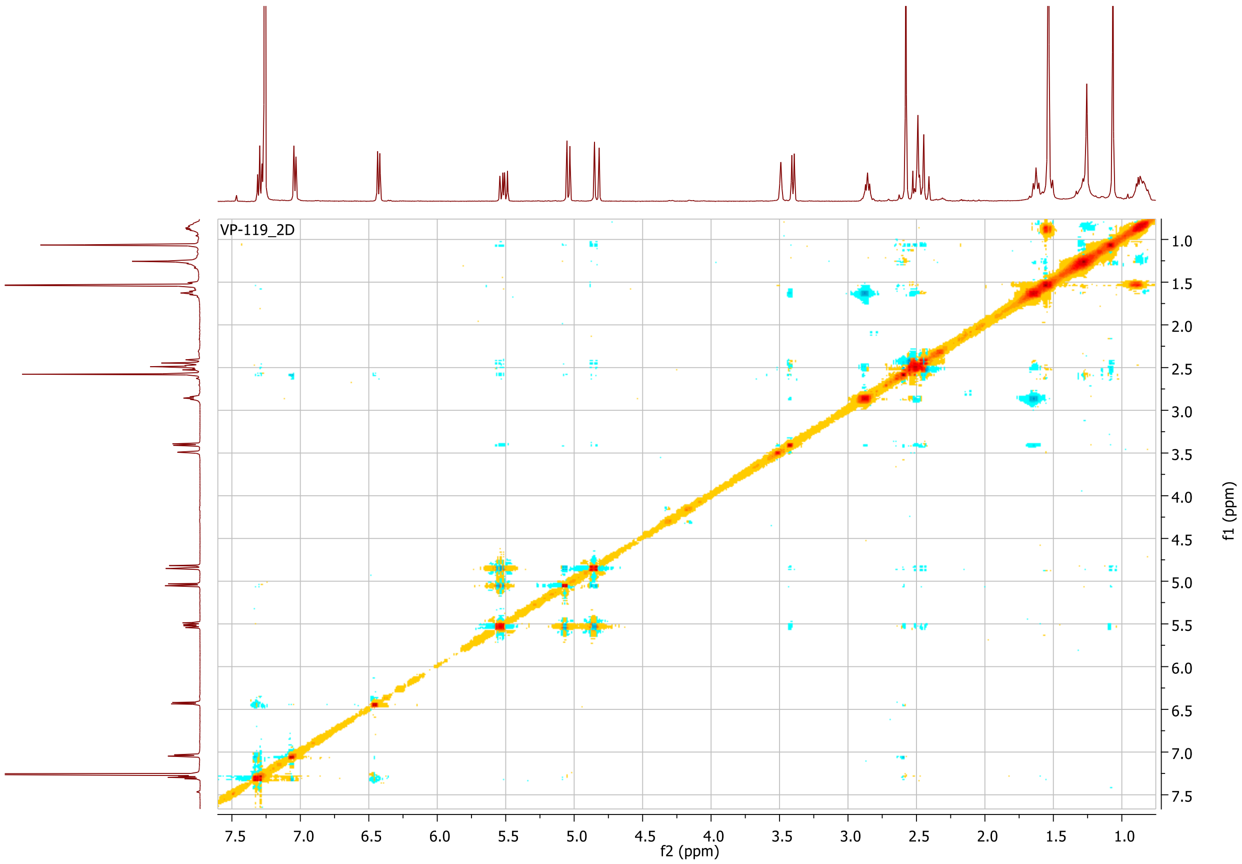

Figure S29. NOESY spectrum of compound **4** ($CDCl_3$, 500 MHz)

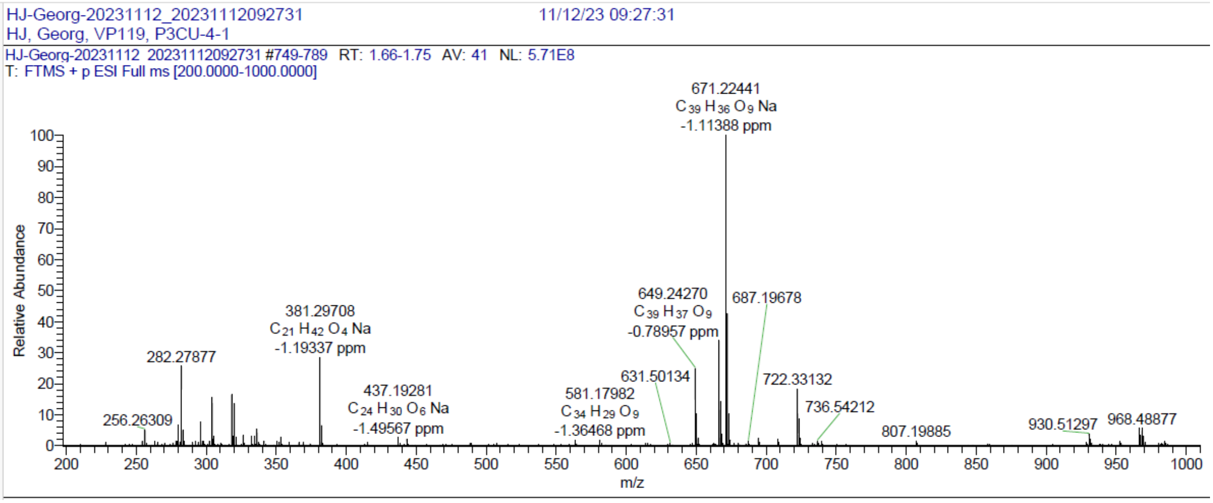

Figure S30. HRESIMS spectrum of compound **4**.

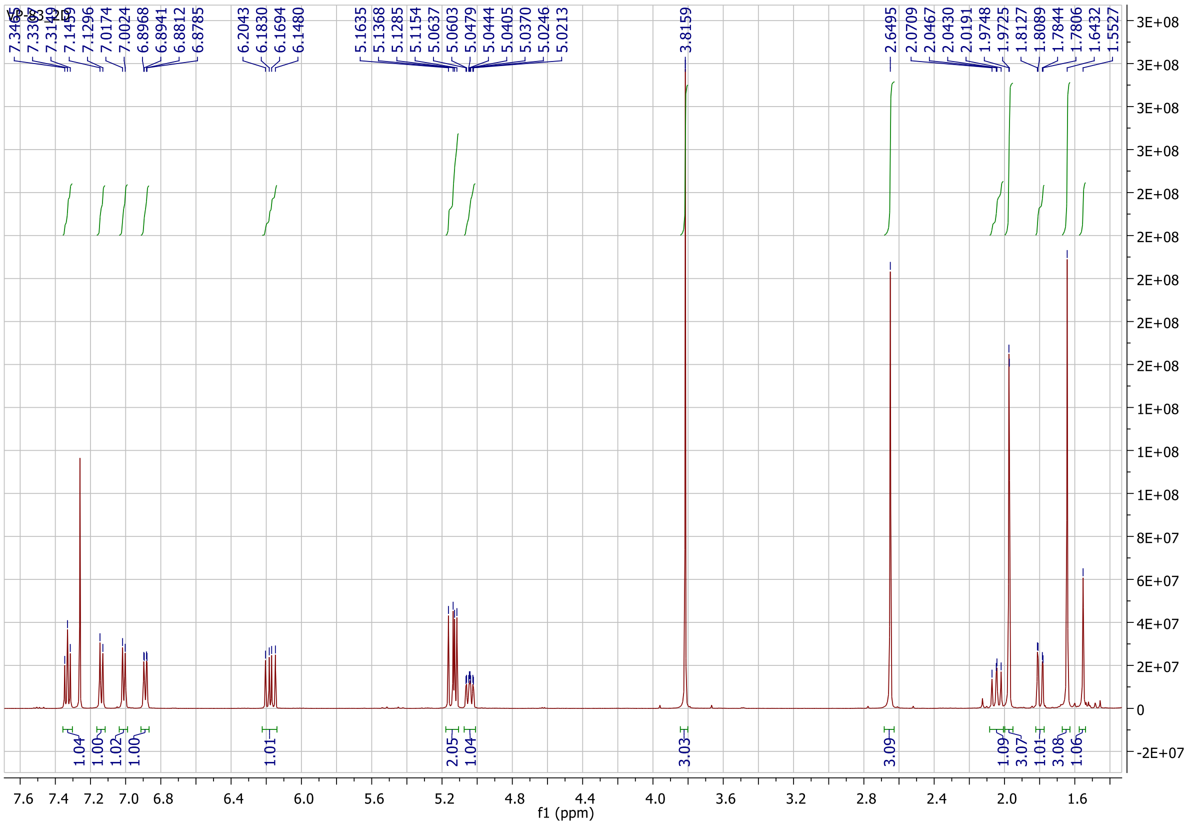

Figure S31. ^{1}H-NMR spectrum of compound **5** ($CDCl_3$, 500 MHz)

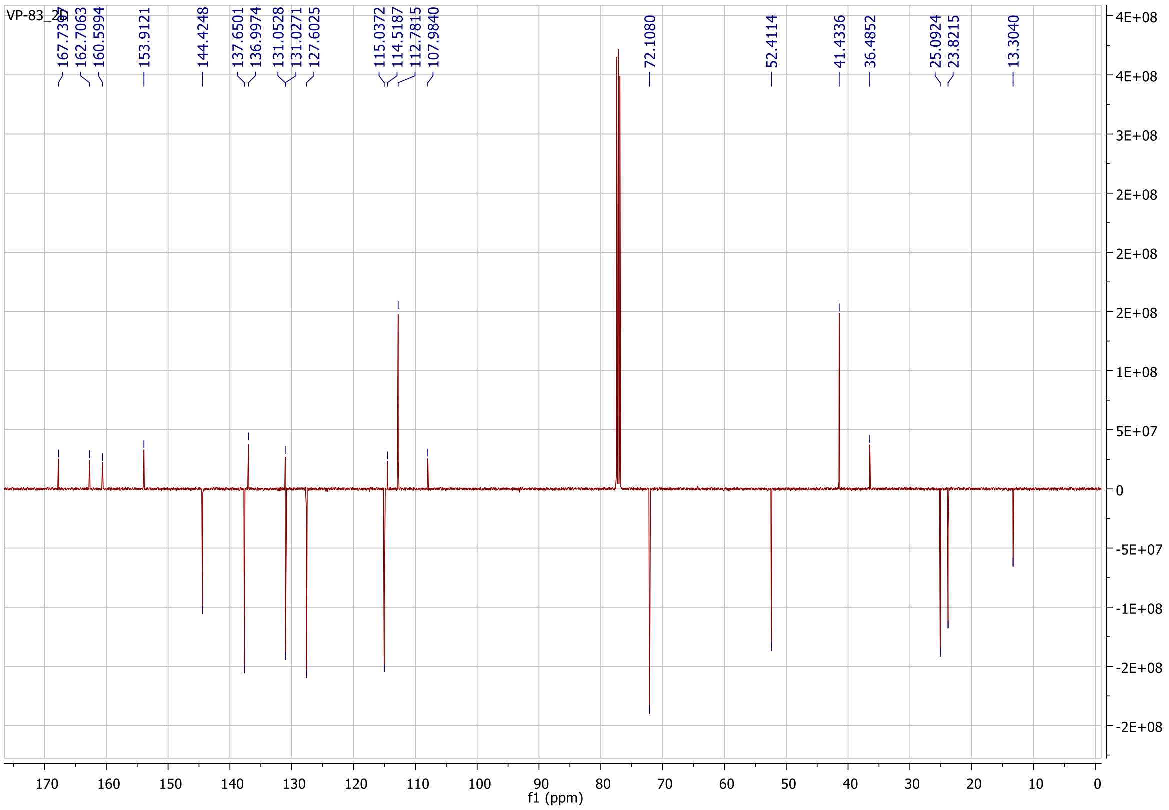

Figure S32. ^{13}C NMR JMOD spectrum of compound **5** ($CDCl_3$, 125 MHz)

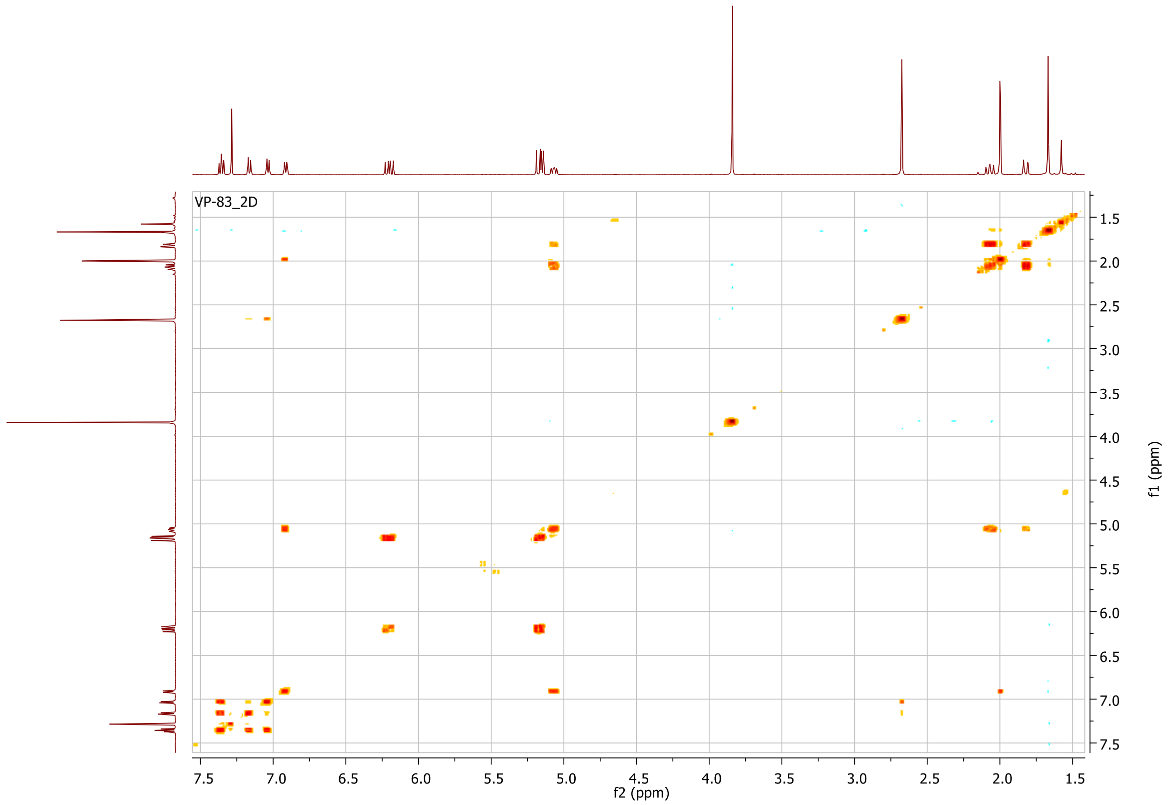

Figure S33. ^{1}H-^{1}H COSY spectrum of compound **5** ($CDCl_3$, 500 MHz)

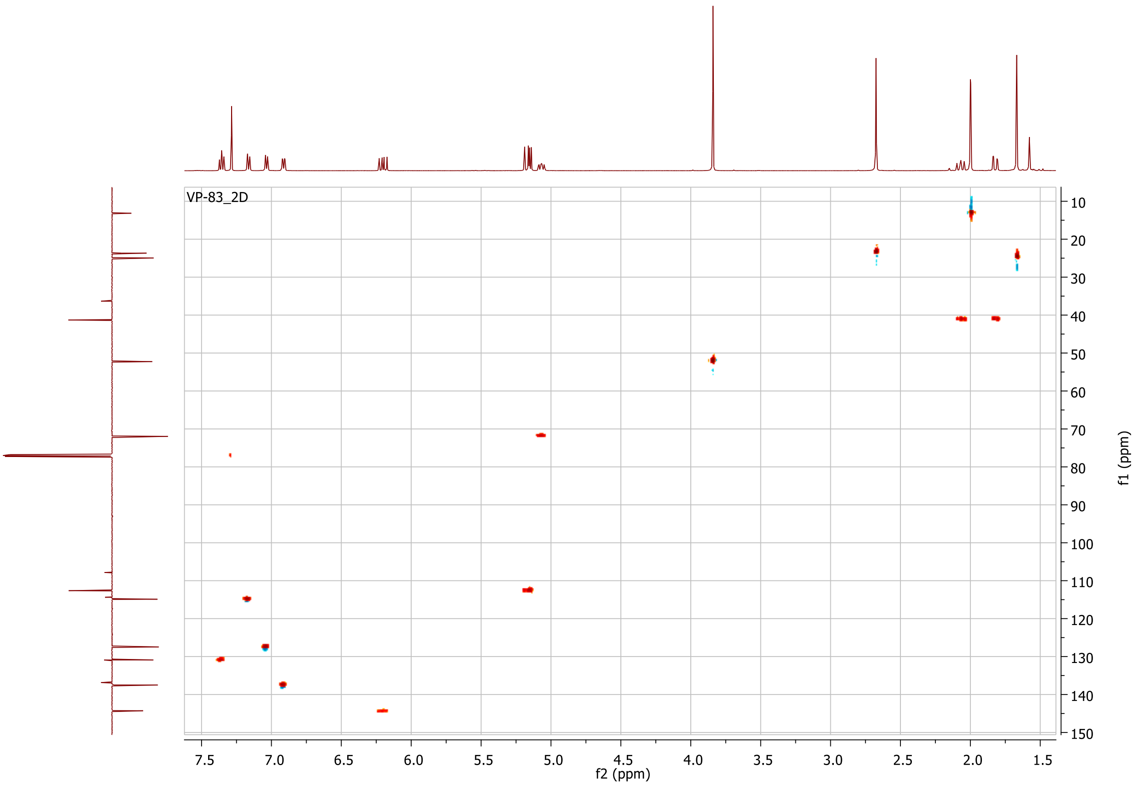

Figure S34. HSQC spectrum of compound **5** ($CDCl_3$, 500/125 MHz)

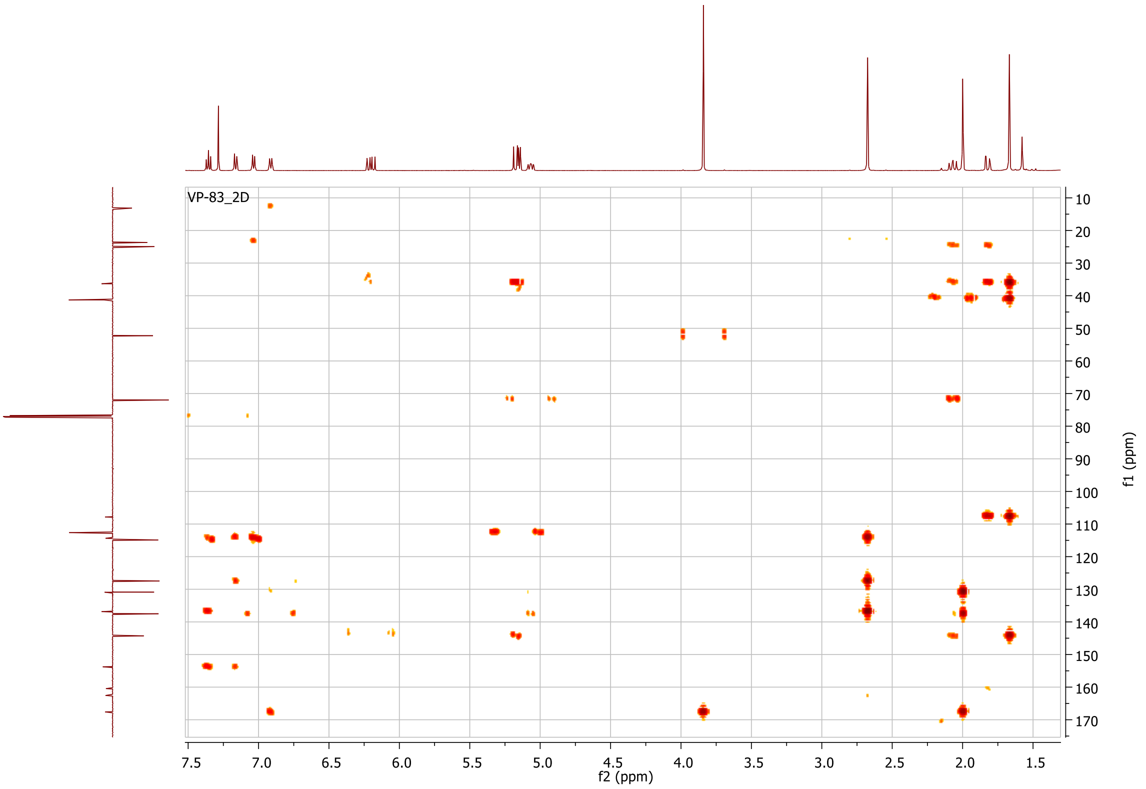

Figure S35. HMBC spectrum of compound **5** ($CDCl_3$, 500/125 MHz)

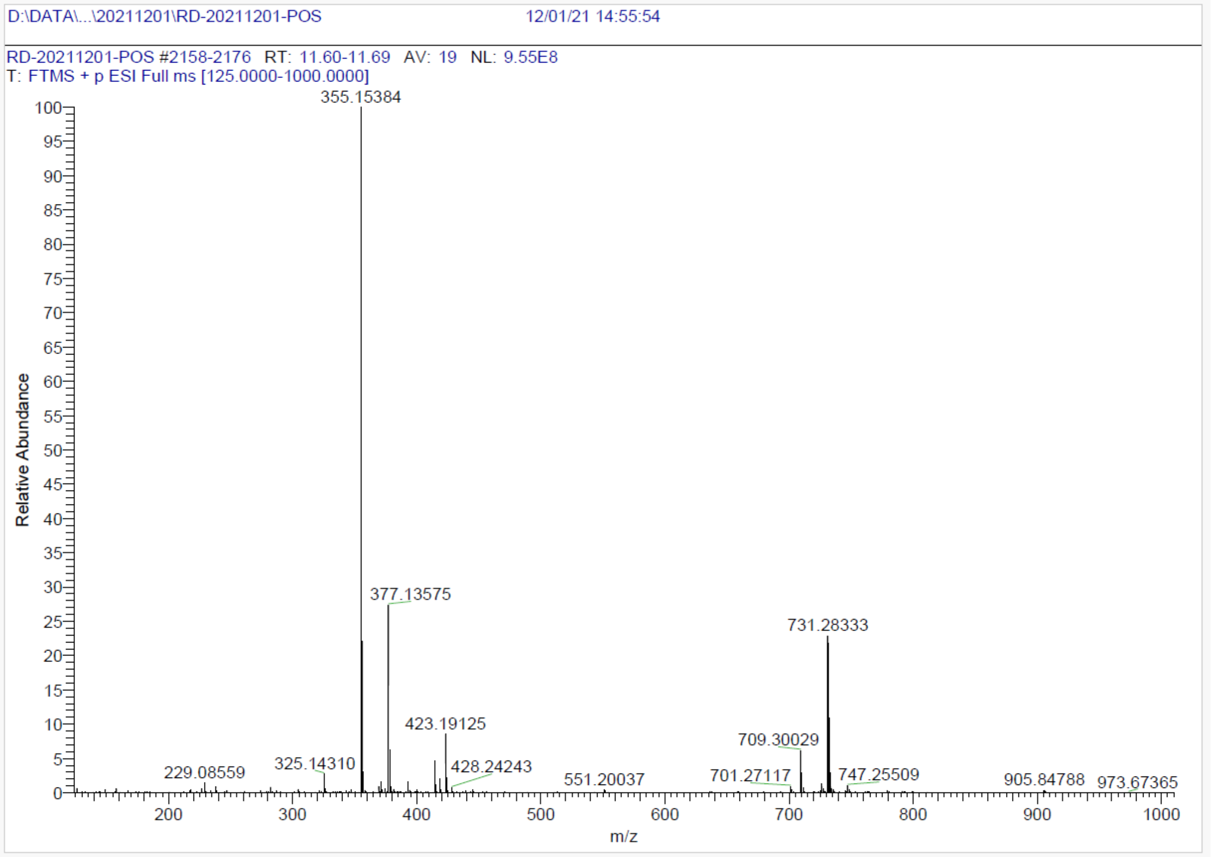

Figure S36. HRESIMS spectrum of compound **5**.

II. STRUCTURE DETERMINATION OF COMPOUND 1 BY SINGLE CRYSTAL X-RAY DIFFRACTION

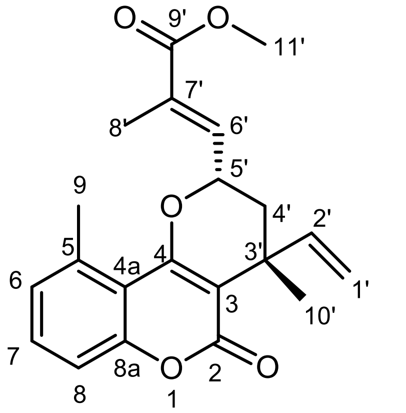

Scheme S1. Formula of (*E)*-methyl 3-(4,10-dimethyl-5-oxo-4-vinyl-2,3,4,5-tetrahydropyrano[3,2-c]chromen-2-yl)-2-methylacrylate **5** (VP83).

The compound (*E)*-methyl 3-(4,10-dimethyl-5-oxo-4-vinyl-2,3,4,5-tetrahydropyrano[3,2-c]chromen-2-yl)-2-methylacrylate **5** (Scheme 1) was isolated from a plant, and crystallized from the mixture of MeOH and ethyl acetate at the Department of Pharmacognosy, Szeged University. In order to improve crystal quality, the compound was recrystallized from the mixture of the same solvents in 3 : 2 ratio by evaporation technique. The single crystal appeared after one day. We have performed several data collections during several weeks. There were crystals kept at room temperature in light. The crystals are stable and proved to be neither heat nor light sensitive.

The reported single crystal X-ray diffraction data collection was performed on a well developed colourless, clear, transparent, block type crystals of **5** (Scheme 2) attached to the loop of the goniometerhead by parathion oil. The aim of the measurement was the determination of the absolute configuration, therefore the single crystal diffraction experiment was performed using CuKα radiation, λ=1.54187Å. The atomic positions were determined by direct methods, all hydrogen atoms were found on the difference Fourier maps [21]. Crystal data and details of the structure determination and refinement are listed in Tables 1, atomic coordinates and equivalent isotropic displacement parameters are in Table 2, hydrogen coordinates and equivalent isotropic displacement parameters are in Table 3, anisotropic displacement parameters are listed in Table 4, bond length and angles, respectively can be found in Table 5 and 6, torsion angles are in Table 7, while intra- and intermolecular interactions of **1** are presented in Table 8.

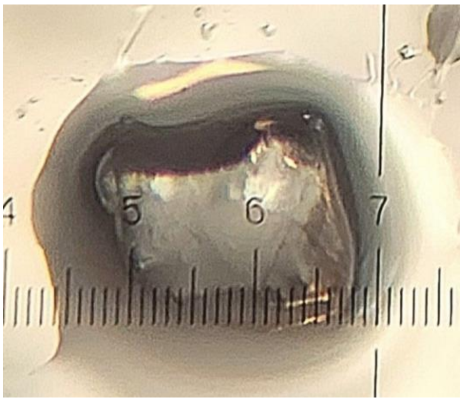

Scheme S2. The measured transparent, block type single crystal of **5** with the size of 0.1 x 0.3 x 0.5 mm under the microscope in paratone oil. 1 full scale is equal to 0.25 mm.

5 crystallizes in the orthorhombic crystal system, in the chiral space group $P2_12_12_1$ (#19). There is one molecule in the asymmetric unit (Figures 1), Z=4, Z'=1. There is no residual solvent accessible void in the crystal lattice, which would be large enough to enclathrate small molecules [Platon]. The C15 carbon atom is chiral, its absolute configuration is *S*. The terminal ethenyl group on C13 is disordered (Figures 2) at room temperature (C11A and C11B with 50-50% occupancy, light grey), while only C11A position can be found with full occupancy at low temperature of -170 °C.

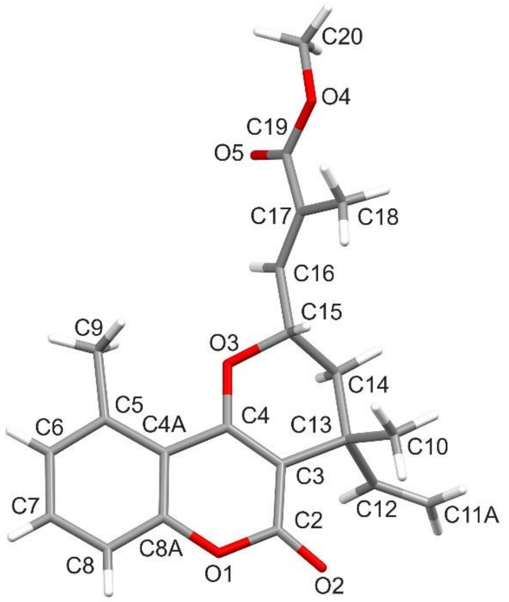

Figure S37. a., Molecular structure diagram of compound **5** showing atom labelling. The absolute configuration of chiral C13 is *R*, and C15 is *S*.

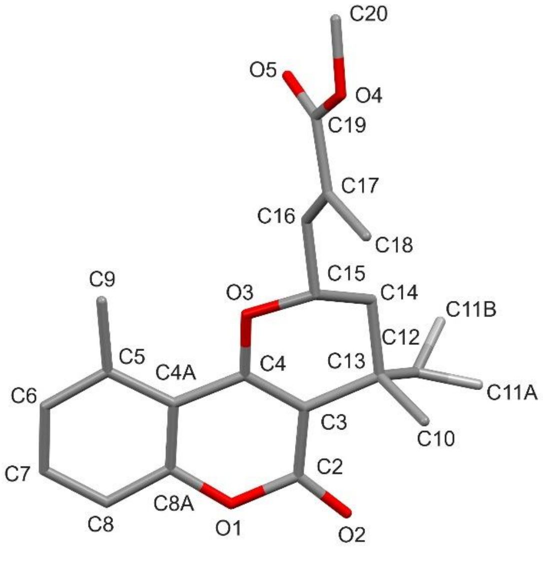

a.,

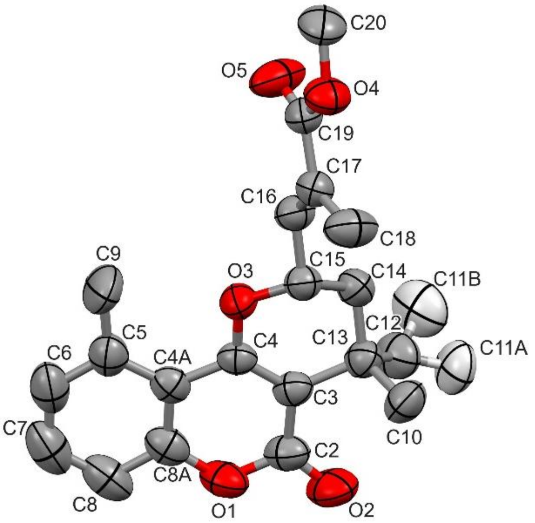

b.,

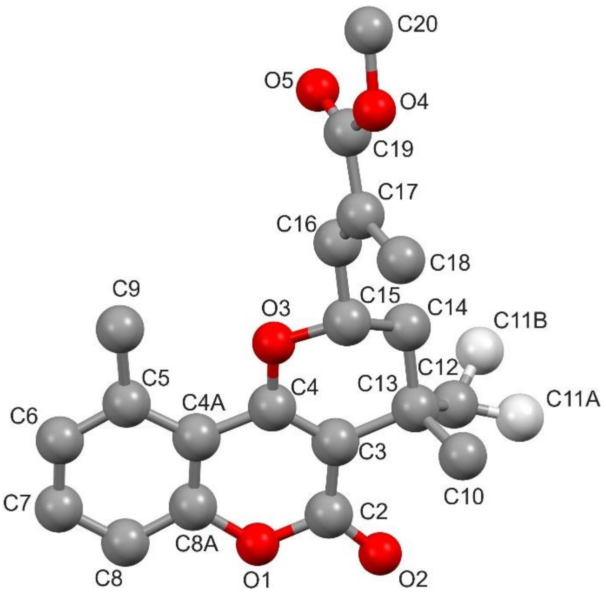

c.,

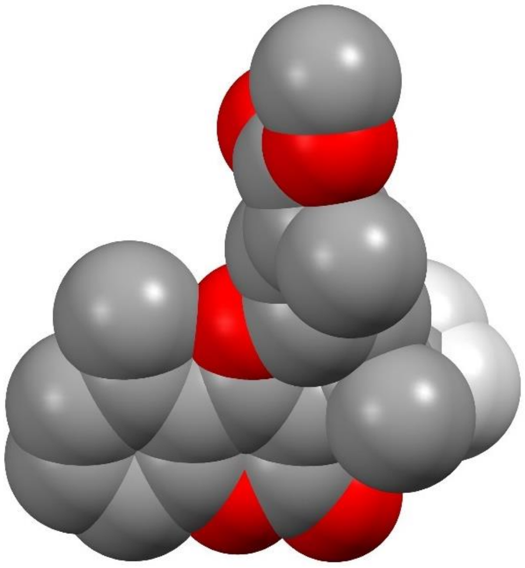

d.,

Figure S38. Molecular structure diagrams of compound **5**. C11 atom of the ethenyl group is disordered at room temperature (light grey) with equal occupancy. Hydrogen atoms are omitted for clarity. a., Capped stick model visualisation. b., ORTEP presentation at 50% probability level. c., Ball and stick representation. d., Space filling model.

The non-aromatic ring contains O3 (sp^3)-C4 (sp^2) -C3 (sp^2) – C13 (sp^3) – C14 (sp^3) – C15 (sp^3) atoms. C14 is 0.309(3)Å, while C15 is -0.324(3)Å out of the plane of this ring. C10 is in axial position (23.54(17)°), C12 is in bisectorial position (48.19(17)°), C16 is in equatorial position (73.57(18)°), while H15 is in axial position (4.97(9)°).

The absolute configuration has been established in the diffraction measurement based on the anomalous-dispersion effect of the five oxygen atoms present in the molecule. The absolute configuration of the chiral C13 is R, and the C15 atom is *S*. The Flack x= 0.09(6) [Flack], Parsons z = 0.03(6). Bijvoet pairs are 1459, Friedel pair coverage 100%. A detailed analysis of the Bijvoet (Friedel) pairs found in an Fo/Fc reflection was performed to establish the absolute structure in terms of the Hooft parameter [Hooft]. Bayesian statistics: P2(true) 1.000, Hooft y= 0.06(5).

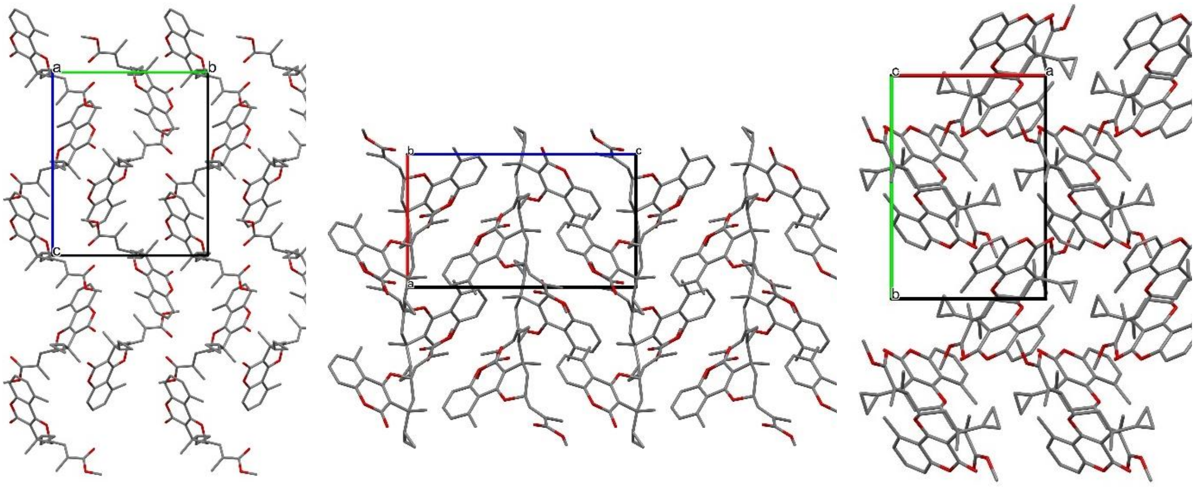

Figure S39. Packing diagram of **5** viewed along the a, b and c crystallographic axes, respectively.

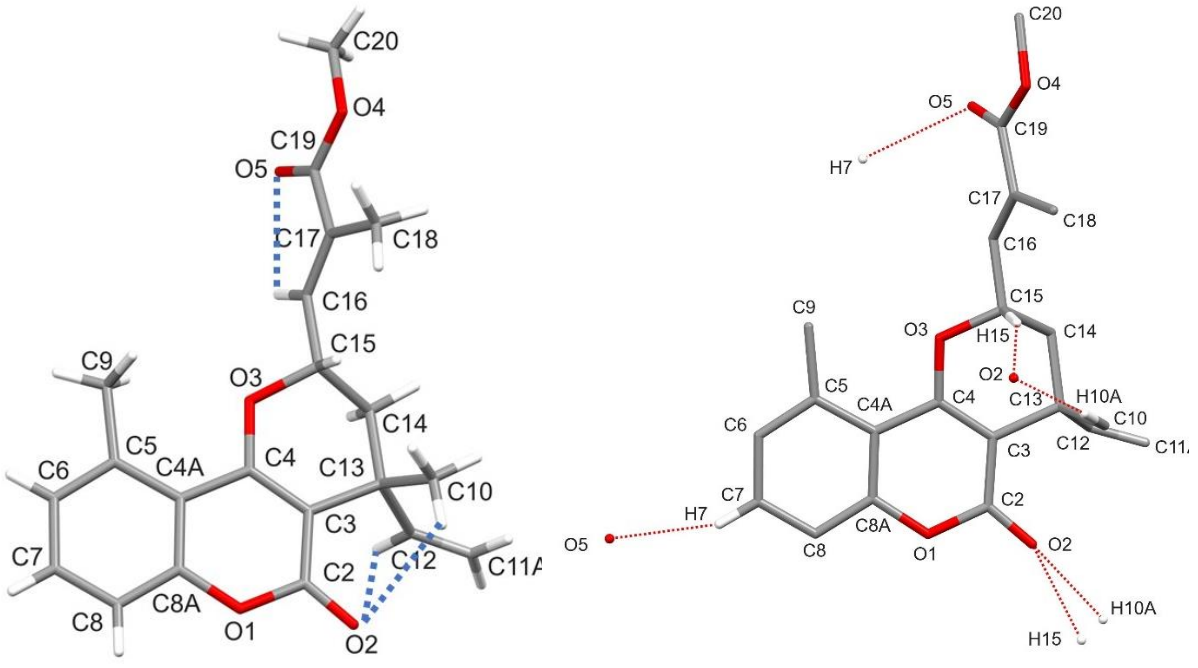

a., b.,

Figure S40. a., Intramolecular and b., intermolecular interactions in the crystal structure of compound **5**.

Packing diagrams viewing from the a, b and c crystallographic axes are presented in Figure 3. No classic hydrogen bonds can be formed in the crystal. In the lack of other donor atoms, only weak C-H…O type interactions can be found in the crystal structure. Three intramolecular interactions contribute to the stability of the molecule (Figure 4a, Table 8). The C2=O2…H15-C15(*) and C2=O2…H10A-C10A intermolecular interactions form infinite chains of molecules in the crystallographic ‘a’ direction. The even weaker C19=O5…H7-C7 interactions connect the molecules in the crystallographic ‘b’ direction (Figure 4b, Table 8).

The calculated morphology of the crystal of **5** is presented in Figure 5 assuming that the growing rates of all crystal sheets are equal. It resembles to the crystal habit received from the single crystal growth experiment.

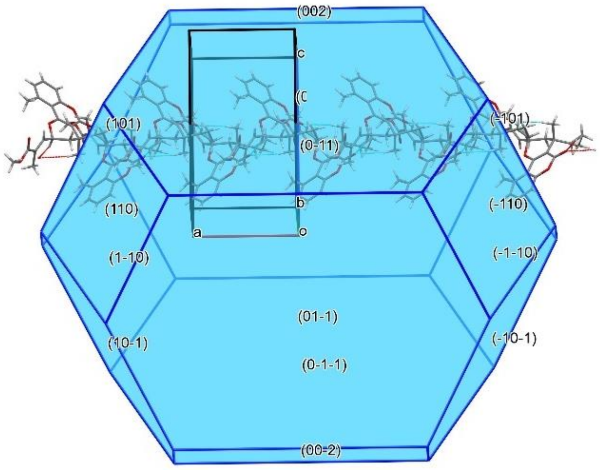

Figure S41. The calculated BFDH morphology of the crystal of **5**.

The powder pattern of **5** is calculated based on the single crystal structure with CuKα radiation in the 5 – 35° 2θ range (Figure 5).

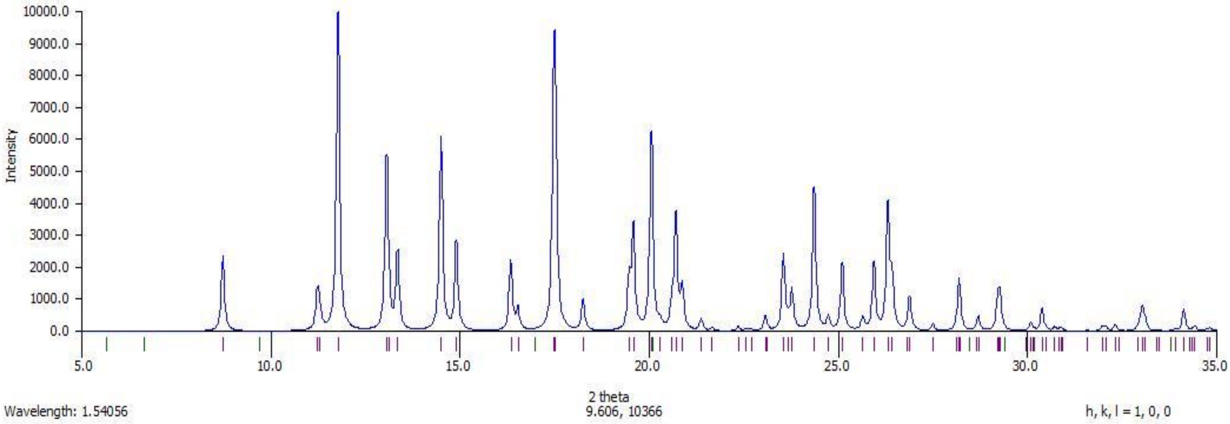

Figure S42. The calculated powder diffraction pattern of **5**.

Experimental

Crystal data: $C_{21}H_{22}O_5$, *Fwt.*: 354.385, colourless, block, size: 0.5 x 0.3 x 0.1 mm, orthorhombic, space group $P2_12_12_1$, a = 9.1089(4)Å, b = 13.2517(6)Å, c = 15.6576(7)Å, α = 90°, β = 90°, γ = 90°,V = 1890.00(15)Å^3, T = 298(2)K, Z = 4, Z' = 1, F(000) = 752, D_x = 1.245 Mg/m^3, μ= 0.724mm^{-1}.

A crystal of **1** was mounted on a loop. Cell parameters were determined by least-squares using 21622 (3.335 ≤ θ ≤ 68.260°) reflections. Intensity data were collected on a 'Rigaku RAXIS-RAPID II' diffractometer (graphite monochromator; Cu-*K*α radiation, λ = 1.54187Å) at 293(2) K in the range 4.371 ≤ θ ≤ 68.167 [CrystalClear]. A total of 24816 reflections were collected of which 3424 were unique [*R*(int) = 0.0374, *R*(σ) = 0.0223]; intensities of 3084 reflections were greater than 2σ(*I*). Completeness is θ = 0.996. A numerical absorption correction was applied to the data (the minimum and maximum transmission factors were 0.931 and 0.973).

The structure was solved by direct methods [21]. Anisotropic full-matrix least-squares

refinement [21] on F^2 for all non-hydrogen atoms yielded $R1 = 0.0437$ and $wR2 = 0.1050$ for 1332 [I>2σ(I)] and $R1 = 0.0489$ and $wR2 = 0.1081$ for all (3424) intensity data, (number of parameters = 249, goodness-of-fit = 1.097, the maximum and mean shift/esd is 0.000 and 0.000). The absolute structure parameter is 0.09(6). (Friedel coverage: 0.742, Friedel fraction max.: 0.995, Friedel fraction full: 0.995).

The maximum and minimum residual electron densities in the final difference map were 0.161 and -0.131e.Å^{-3}. The weighting scheme applied was $w = 1/[\sigma^2(F_o^2) + (0.0589P)^2 +0.0865P]$, where $P = (F_o^2+ 2F_c^2)/3$.

Hydrogen atomic positions were located in difference maps, then they were constrained. Hydrogen atoms were included in structure factor calculations but they were not refined. The isotropic displacement parameters of the hydrogen atoms were approximated from the U(eq) value of the atom they were bonded to.

Table S1. Crystal data and details of the structure determination and refinement of **5**

Empirical formula	$C_{21}H_{22}O_5$
Formula weight	354.385
Temperature	298(2)
Radiation and wavelength	Cu-Kα, λ =1.54187Å
Crystal system	orthorhombic
Space group	$P2_12_12_1$
Unit cell dimensions	a =9.1089(4)Å, b =13.2517(6)Å, c =15.6576(7)Å; α = β = γ = 90°
Volume	1890.00(15)Å^3
Z	4
Density (calculated)	1.245 Mg/m^3
Absorption coefficient, μ	0.724 mm^{-1}
F(000)	752
Crystal colour	colourless
Crystal description	block
Crystal size	0.5 x 0.3 x 0.1 mm
Absorption correction	numerical
Max. and min. transmission	0.931 0.973
θ–range for data collection	4.371 ≤ θ ≤ 68.167°
Index ranges	$-10 \le h \le 10$; $-15\le k \le 15$; $-18 \le l \le 17$
Reflections collected	24816
Completeness to 2θ	0.996
Absolute structure parameter	0.09(6)
Friedel coverage	0.742
Friedel fraction max.	0.995
Friedel fraction full	0.995
Independent reflections	3424 [R(int) =0.0374]
Reflections I>2σ(I)	3084
Refinement method	full-matrix least-squares on F^2
Data / restraints / parameters	3424 /2 /249

Goodness-of-fit on F^2	1.097
Final *R* indices [$I>2\sigma(I)$]	*R*1 =0.0437, *wR*2 =0.1050
R indices (all data)	*R*1 =0.0489, *wR*2 =0.1081
Max. and mean shift/esd	0.000; 0.000
Largest diff. peak and hole	0.161; -0.131 e.Å^{-3}

Table S2. Atomic coordinates and equivalent isotropic displacement parameters of **5**. *U*(eq) is defined as one third of the trace of the orthogonalized U_{ij} tensor.

Atom	x	y	z	U(eq) [Å^2]
O1	0.1212(2)	0.23096(15)	0.68707(15)	0.0824(8)
O2	-0.0468(3)	0.22992(17)	0.58861(18)	0.1007(9)
O3	0.34755(18)	0.46096(13)	0.57348(11)	0.0633(6)
O4	0.6215(2)	0.71210(15)	0.31956(13)	0.0775(7)
O5	0.5328(3)	0.76519(17)	0.44287(14)	0.1055(9)
C2	0.0628(3)	0.2714(2)	0.6142(2)	0.0737(10)
C3	0.1368(3)	0.35535(17)	0.57556(18)	0.0593(8)
C4	0.2650(3)	0.38892(18)	0.60943(16)	0.0555(8)
C4A	0.3226(3)	0.34904(18)	0.68897(17)	0.0590(8)
C5	0.4500(3)	0.3826(2)	0.73264(19)	0.0731(10)
C6	0.4860(4)	0.3360(3)	0.8088(2)	0.0903(12)
C7	0.4049(5)	0.2584(3)	0.8430(2)	0.1000(16)
C8	0.2838(4)	0.2239(3)	0.8014(2)	0.0890(12)
C8A	0.2445(3)	0.2695(2)	0.72527(19)	0.0692(10)
C9	0.5466(4)	0.4671(3)	0.7025(3)	0.1110(16)
C10	0.0770(4)	0.3271(2)	0.4202(2)	0.0835(11)
*C11A	-0.1997(6)	0.4224(7)	0.4689(5)	0.097(3)
*C11B	-0.1594(9)	0.5124(7)	0.5121(6)	0.120(4)
C12	-0.0902(3)	0.4305(3)	0.5133(2)	0.0878(13)
C13	0.0684(3)	0.4018(2)	0.49519(18)	0.0639(9)
C14	0.1535(3)	0.49771(19)	0.47412(19)	0.0657(9)
C15	0.3168(3)	0.48417(18)	0.48452(16)	0.0586(8)
C16	0.3995(3)	0.57812(19)	0.46515(18)	0.0646(9)
C17	0.4802(3)	0.59718(18)	0.39730(16)	0.0591(8)
C18	0.5045(5)	0.5251(2)	0.3250(2)	0.0931(13)
C19	0.5459(3)	0.6999(2)	0.39094(18)	0.0648(9)
C20	0.6830(4)	0.8106(2)	0.3049(2)	0.0923(12)

U(eq) = 1/3 of the trace of the orthogonalized U Tensor

Starred Atom sites have a S.O.F less than 1.0

Table S3. Hydrogen coordinates and isotropic displacement parameters of 5.

Atom	x	y	z	U(iso) [Å²]
H6	0.56871	0.35805	0.83812	0.1090
H7	0.43281	0.22951	0.89467	0.1200
H8	0.22874	0.17085	0.82349	0.1070
H9A	0.59021	0.44914	0.64880	0.1660
H9B	0.48894	0.52718	0.69561	0.1660
H9C	0.62248	0.47900	0.74385	0.1660
H10A	0.17721	0.30730	0.41141	0.1250
H10B	0.04018	0.35883	0.36937	0.1250
H10C	0.01883	0.26860	0.43291	0.1250
*H11A	-0.19256	0.39490	0.41440	0.1160
*H11B	-0.29020	0.44382	0.48952	0.1160
*H11C	-0.11128	0.57191	0.49767	0.1800
*H11D	-0.25882	0.51360	0.52562	0.1800
*H12A	-0.10642	0.45868	0.56684	0.1050
*H12B	-0.14834	0.37563	0.52846	0.1050
H14A	0.13255	0.51739	0.41571	0.0790
H14B	0.12058	0.55172	0.51131	0.0790
H15	0.35124	0.42910	0.44787	0.0700
H16	0.39351	0.62933	0.50562	0.0780
H17A	0.46601	0.46005	0.34008	0.1390
H17B	0.60777	0.51949	0.31378	0.1390
H17C	0.45531	0.54956	0.27492	0.1390
H20A	0.72534	0.81302	0.24876	0.1380
H20B	0.75770	0.82384	0.34667	0.1380
H20C	0.60715	0.86056	0.30941	0.1380

The Temperature Factor has the Form of Exp(-T) Where
T = 8*(Pi**2)*U*(Sin(Theta)/Lambda)**2 for Isotropic Atoms

Table S4. Anisotropic displacement parameters of **1**. The anisotropic displacement factor exponent takes the form: $-2\pi^2(h^2a^{*2}U_{11} + ... + 2hka^*b^*U_{12})$.

Atom	U(1,1) or U	U(2,2)	U(3,3)	U(2,3)	U(1,3)	U(1,2)
O1	0.0787(13)	0.0644(11)	0.1041(16)	0.0239(11)	0.0099(12)	-0.0056(10)
O2	0.0803(14)	0.0749(13)	0.147(2)	0.0128(14)	-0.0077(15)	-0.0258(11)
O3	0.0599(9)	0.0642(10)	0.0658(11)	0.0075(8)	-0.0054(8)	-0.0124(8)
O4	0.0870(13)	0.0689(11)	0.0766(13)	0.0116(10)	0.0135(10)	-0.0128(10)
O5	0.156(2)	0.0754(13)	0.0852(14)	-0.0143(12)	0.0207(15)	-0.0473(15)
C2	0.0632(15)	0.0560(14)	0.102(2)	0.0037(15)	0.0045(15)	-0.0041(13)
C3	0.0562(13)	0.0503(12)	0.0715(16)	-0.0032(12)	0.0029(12)	0.0001(11)
C4	0.0574(13)	0.0459(12)	0.0633(15)	-0.0010(11)	0.0065(11)	0.0020(10)
C4A	0.0633(14)	0.0548(13)	0.0590(15)	-0.0023(11)	0.0068(12)	0.0102(11)
C5	0.0756(16)	0.0709(16)	0.0728(18)	-0.0092(14)	-0.0080(15)	0.0133(14)
C6	0.100(2)	0.098(2)	0.073(2)	-0.0106(18)	-0.0194(18)	0.029(2)
C7	0.125(3)	0.107(3)	0.068(2)	0.010(2)	0.003(2)	0.047(3)
C8	0.101(2)	0.082(2)	0.084(2)	0.0220(18)	0.021(2)	0.0265(19)
C8A	0.0717(17)	0.0617(15)	0.0743(18)	0.0036(15)	0.0138(14)	0.0161(14)
C9	0.093(2)	0.119(3)	0.121(3)	0.011(2)	-0.045(2)	-0.031(2)
C10	0.0813(19)	0.0803(19)	0.089(2)	-0.0174(16)	-0.0124(17)	-0.0105(16)
C11A	0.056(3)	0.121(7)	0.114(6)	-0.009(5)	-0.018(4)	0.006(3)
C11B	0.074(4)	0.134(8)	0.152(8)	0.017(6)	0.017(5)	0.020(5)
C12	0.0615(17)	0.091(2)	0.111(3)	0.010(2)	-0.0002(17)	0.0048(16)
C13	0.0557(13)	0.0602(14)	0.0759(18)	-0.0025(13)	-0.0070(12)	-0.0013(12)
C14	0.0650(15)	0.0587(14)	0.0733(17)	0.0035(13)	-0.0064(13)	0.0043(12)
C15	0.0631(14)	0.0527(13)	0.0599(16)	-0.0002(11)	0.0011(12)	-0.0033(11)
C16	0.0723(16)	0.0542(14)	0.0674(16)	-0.0026(13)	0.0019(13)	-0.0068(12)
C17	0.0625(14)	0.0545(13)	0.0603(15)	0.0030(11)	-0.0006(12)	-0.0011(11)
C18	0.127(3)	0.0644(18)	0.088(2)	-0.0064(15)	0.023(2)	-0.0107(18)
C19	0.0690(15)	0.0605(15)	0.0649(17)	0.0058(13)	-0.0083(14)	-0.0092(12)
C20	0.100(2)	0.084(2)	0.093(2)	0.0271(18)	0.0041(19)	-0.0270(18)

===

The Temperature Factor has the Form of Exp(-T) Where
T = 8*(Pi**2)*U*(Sin(Theta)/Lambda)**2 for Isotropic Atoms
T = 2*(Pi**2)*Sumij(h(i)*h(j)*U(i,j)*Astar(i)*Astar(j)), for Anisotropic Atoms. Astar(i) are Reciprocal Axial Lengths and h(i) are the Reflection Indices.

Table S5. Bond lengths [Å] of **5**.

O1-C2	1.368(4)	O1-C8a	1.371(4)
O2-C2	1.208(4)	C2-C3	1.435(4)
O3-C4	1.339(3)	O3-C15	1.454(3)
C3-C4	1.357(3)	C3-C13	1.533(4)
O4-C19	1.323(3)	O4-C20	1.439(3)
C4-C4a	1.451(3)	O5-C19	1.193(3)
C5-C6	1.382(4)	C5-C4a	1.418(4)
C5-C9	1.500(5)	C6-C7	1.375(5)
C7-C8	1.361(5)	C8-C8a	1.383(4)
C10-C13	1.538(4)	C12-C11b	1.256(8)
C12-C11a	1.220(1)	C12-C13	1.521(4)
C13-C14	1.525(4)	C14-C15	1.507(4)
C15-C16	1.487(3)	C16-C17	1.316(4)
C17-C19	1.490(3)	C17-C18	1.497(4)
C4a-C8a	1.393(4)		

Table S6. Bond angles [°] of **5**.

C2-O1-C8a	122.4(2)	O2-C2-O1	114.8(3)
O2-C2-C3	127.0(3)	O1-C2-C3	118.2(2)
C4-O3-C15	116.4(2)	C4-C3-C2	119.6(3)
C4-C3-C13	122.6(2)	C2-C3-C13	117.8(2)
C19-O4-C20	116.7(2)	O3-C4-C3	123.5(2)
O3-C4-C4a	114.7(2)	C3-C4-C4a	121.8(2)
C6-C5-C4a	118.0(3)	C6-C5-C9	117.7(3)
C4a-C5-C9	124.2(3)	C7-C6-C5	122.9(4)
C8-C7-C6	120.0(3)	C7-C8-C8a	118.4(4)
C11b-C12-C13	133.6(5)	C11a-C12-C13	130.4(5)
C12-C13-C14	108.4(2)	C12-C13-C3	109.5(2)
C14-C13-C3	107.8(2)	C12-C13-C10	110.6(2)
C14-C13-C10	110.2(2)	C3-C13-C10	110.3(2)
C15-C14-C13	112.3(2)	O3-C15-C16	106.0(2)
O3-C15-C14	108.6(2)	C16-C15-C14	112.2(2)
C17-C16-C15	127.5(2)	C16-C17-C19	117.0(2)
C16-C17-C18	124.8(2)	C19-C17-C18	118.2(2)
O5-C19-O4	122.6(2)	O5-C19-C17	125.2(3)
O4-C19-C17	112.2(2)	C8a-C4a-C5	117.3(3)
C8a-C4a-C4	116.2(2)	C5-C4a-C4	126.5(2)
O1-C8a-C8	115.1(3)	O1-C8a-C4a	121.5(3)
C8-C8a-C4a	123.4(3)		

Table S7. Torsion angles [°] of **5**.

C8a-O1-C2-O2	-179.3(3)	C8a-O1-C2-C3	1.9(4)
O2-C2-C3-C4	-175.5(3)	O1-C2-C3-C4	3.1(4)
O2-C2-C3-C13	2.9(4)	O1-C2-C3-C13	-178.5(2)
C15-O3-C4-C3	-16.2(3)	C15-O3-C4-C4a	164.29(19)
C2-C3-C4-O3	174.2(2)	C13-C3-C4-O3	-4.1(4)
C2-C3-C4-C4a	-6.3(4)	C13-C3-C4-C4a	175.4(2)
C4a-C5-C6-C7	0.7(5)	C9-C5-C6-C7	179.4(3)
C5-C6-C7-C8	0.5(5)	C6-C7-C8-C8a	-0.8(5)
C11b-C12-C13-C14	1.9(8)	C11a-C12-C13-C14	102.8(7)
C11b-C12-C13-C3	119.2(7)	C11a-C12-C13-C3	-139.8(7)
C11b-C12-C13-C10	-119.0(7)	C11a-C12-C13-C10	-18.1(7)
C4-C3-C13-C12	-127.4(3)	C2-C3-C13-C12	54.2(3)
C4-C3-C13-C14	-9.7(3)	C2-C3-C13-C14	171.9(2)
C4-C3-C13-C10	110.7(3)	C2-C3-C13-C10	-67.7(3)
C12-C13-C14-C15	160.5(2)	C3-C13-C14-C15	42.1(3)
C10-C13-C14-C15	-78.4(3)	C4-O3-C15-C16	168.9(2)
C4-O3-C15-C14	48.2(3)	C13-C14-C15-O3	-62.4(3)
C13-C14-C15-C16	-179.2(2)	O3-C15-C16-C17	134.9(3)
C14-C15-C16-C17	-106.7(3)	C15-C16-C17-C19	176.9(3)
C15-C16-C17-C18	-0.2(5)	C20-O4-C19-O5	-2.7(4)
C20-O4-C19-C17	176.8(2)	C16-C17-C19-O5	1.1(4)
C18-C17-C19-O5	178.4(3)	C16-C17-C19-O4	-178.3(2)
C18-C17-C19-O4	-1.0(4)	C6-C5-C4a-C8a	-1.4(4)
C9-C5-C4a-C8a	180.0(3)	C6-C5-C4a-C4	178.9(2)
C9-C5-C4a-C4	0.3(4)	O3-C4-C4a-C8a	-176.0(2)
C3-C4-C4a-C8a	4.4(3)	O3-C4-C4a-C5	3.7(4)
C3-C4-C4a-C5	-175.9(2)	C2-O1-C8a-C8	176.1(2)
C2-O1-C8a-C4a	-3.8(4)	C7-C8-C8a-O1	-179.8(3)
C7-C8-C8a-C4a	0.1(4)	C5-C4a-C8a-O1	-179.0(2)
C4-C4a-C8a-O1	0.7(3)	C5-C4a-C8a-C8	1.1(4)
C4-C4a-C8a-C8	-179.2(2)		

Table S8. Intra- and intermolecular interactions in **5**.

D-H…A	D-H [Å]	H…A [Å]	D…A [Å]	D-H…A [°]	symmetry operation
C10-H10C...O2	0.96	2.56	3.144(4)	119	Intra
C12-H12B...O2	0.93	2.34	2.935(5)	122	Intra
C16-H16...O5	0.93	2.41	2.782(3)	104	intra
C10-H10A...O2	0.96	2.56	3.512(5)	170	1/2+x,1/2-y,1-z
C15-H15...O2	0.98	2.37	3.302(3)	158	1/2+x,1/2-y,1-z
C7-H7...O5	0.93	2.61	3.402(4)	130	1-x,1/2+y,3/2-z

III. References

20. CrystalClear SM 1.4.0 (Rigaku/MSC Inc., 2008).

21. Sheldrick, G.M. *Acta Cryst*. (2008) A64, 112-122.

27. Flack, H. D. *Acta Cryst*. (1983) A39, 876-881.

28. Hooft, R.W.W.; Straver, L.H.; Spek, A.L. *J. Appl. Cryst*. (2008), 41, 96-103.

29. Higashi, T. Numerical Absorption Correction, NUMABS, 2002

30. Spek, A. L. (2015). *Acta Cryst.* C71, 9-18.

IV. Figure S43. Isolation flow chart

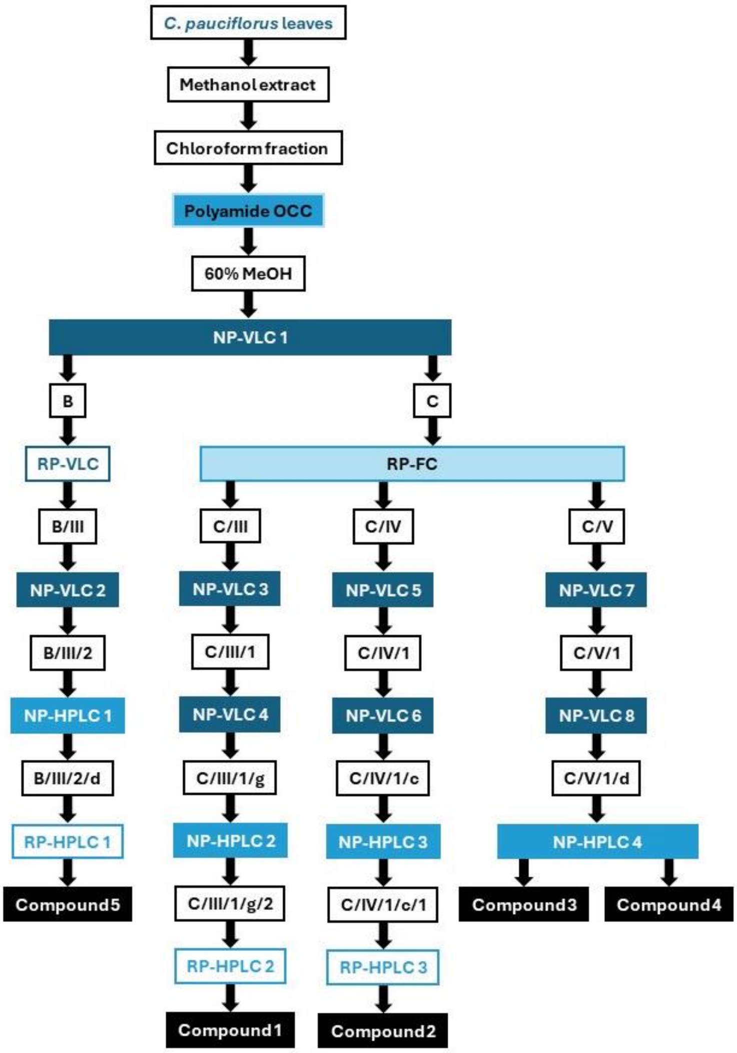

Techniques	Solvents	Mode
Polyamide OCC	methanol – water	gradient
RP-FC	methanol – water	gradient
NP-VLC 1	cyclohexane – ethyl acetate – ethanol	gradient
NP-VLC 2	cyclohexane – ethyl acetate	gradient
NP-VLC 3	*n*-hexane – chloroform	gradient
NP-VLC 4	cyclohexane – ethyl acetate	gradient
NP-VLC 5	*n*-hexane – chloroform	gradient
NP-VLC 6	cyclohexane – ethyl acetate	gradient
NP-VLC 7	*n*-hexane – chloroform	gradient
NP-VLC 8	cyclohexane – ethyl acetate	gradient
RP-VLC	methanol – water	gradient
NP-HPLC 1	*n*-hexane – ethyl acetate – methanol (98:1:1)	isocratic
NP-HPLC 2	*n*-hexane – ethyl acetate – methanol (80:19:1)	isocratic
NP-HPLC 3	*n*-hexane – ethyl acetate – methanol (80:19:1)	isocratic
NP-HPLC 4	*n*-hexane – ethyl acetate – methanol (80:19:1)	isocratic
RP-HPLC 1	methanol – water (75:25)	isocratic
RP-HPLC 2	methanol – water (75:25)	isocratic
RP-HPLC 3	methanol – water (8:2)	isocratic
RP-HPLC 3	methanol – water (67:33)	isocratic
